# The role of between-group signaling in the evolution of primate ornamentation

**DOI:** 10.1093/evlett/qrae045

**Published:** 2024-08-17

**Authors:** Cyril C Grueter, Stefan Lüpold

**Affiliations:** Department of Anatomy, Physiology and Human Biology, School of Human Sciences, The University of Western Australia, Perth, WA, Australia; International Centre of Biodiversity and Primate Conservation, Dali University, Dali, China; Centre for Evolutionary Biology, School of Biological Sciences, The University of Western Australia, Perth, WA, Australia; Centre of Excellence in Biodiversity and Natural Resource Management, University of Rwanda, Huye, Rwanda; Department of Evolutionary Biology and Environmental Studies, University of Zurich, Zurich, Switzerland

**Keywords:** sexual selection, ornaments, primates, signals, inter-group interactions, home range overlap

## Abstract

Gregarious mammals interact to varying degrees and in a variety of ways with neighboring groups. Since navigating this wider social environment via conventional means (social knowledge) may be challenging, we hypothesize that between-group socio-spatial dynamics have exerted strong selection on phenotypic markers of individual identity, quality, and competitive ability. Ornaments are sexually selected decorative traits with far-reaching signaling potential. Here, we examined the links between sexual dimorphism in ornamentation, home range use and encounter rates across 144 primate species in a Bayesian framework. We show that home range overlap (shared space among neighbors), an indicator of the complexity of between-group interactions (but not necessarily male-male competition), is positively associated with dimorphism in ornamentation. We find no clear effect for between-group encounter rates. We also find that inter-group interactions were less agonistic when there was greater home range overlap. Taken together, these findings indicate that ornaments play a hitherto underappreciated role in signaling to conspecifics outside the realms of their home groups.

## Introduction

In gregarious species, social groups often overlap in their home ranges and so are likely to encounter and interact with other groups (Christensen & Radford, 2018; Van Belle et al., 2020; Waser, 1976; Willems et al., 2013). Interactions can range from peaceful to hostile, depending on the relative competitive abilities of interactants, interaction location, nature of the resources at stake, anticipated payoffs, or the relatedness and familiarity between groups (Cheney, 1987; Crofoot et al., 2008; Grueter & White, 2014; Grueter et al., 2016; Majolo et al., 2020; Mirville et al., 2018; Pisor & Surbeck, 2019; Reichard & Sommer, 1997; Strong et al., 2018). While interactions can be predictable within a set of neighbors in territorial systems, they may not be when the overlap of home ranges is substantial (Grueter, 2015). Between-group social dynamics can exert strong selection on within-group affiliation and cohesion (Bruintjes et al., 2016; Mirville et al., 2020; Radford, 2008; but see Grueter, 2013), competitive regimes (Sterck et al., 1997), bondedness (Wrangham, 1980), collective action and border patrols (Mitani & Watts, 2005), space use (Brown, 2020; Markham et al., 2013), or vigilance and sentinel behavior (Gaynor & Cords, 2012; Morris-Drake et al., 2019). Further, between-group interactions can influence the evolution of morphological and physiological traits, including increasingly larger male relative to female body size as the frequency and intensity of inter-group competition increase (Grueter & van Schaik, 2009). Similarly, interactions between social groups can mold the composition of the gut microbiota (Wikberg et al., 2020), and the challenges and opportunities associated with between-group interactions have been hypothesized to foster cognitive evolution (Ashton et al., 2020). Indeed, there is empirical evidence that navigating the wider beyond-group social sphere requires investments in cognitive capital (i.e., brain size; (Grueter, 2015)).

Between-group/intruder pressures are also thought to affect the differentiation of markers of individual identity, quality and competitive ability, such as ornaments (Grueter, 2021). Ornaments or phenotypic accouterments, including hairy, fleshy and colorful secondary sexual traits, have evolved for a variety of purposes, including species recognition/discrimination (Caro et al., 2021; Santana et al., 2012; Winters et al., 2020) and intra-specific social signaling (Bergman et al., 2009; Gerald, 2001; Grueter et al., 2015; Marty et al., 2009). Here, we focus on intra-species social signaling, with a particular emphasis on the role of sexual selection in shaping the evolution of secondary sexual ornaments. Some of the best-known examples of sexually selected ornaments include the red chest patches of geladas (*Theropithecus gelada*), the cheek flanges of orangutans (*Pongo* spp.), the hair capes of hamadryas baboons (*Papio hamadryas*), the elongated and thick noses of proboscis monkeys (*Nasalis larvatus*), bright genital coloration in some guenons and the beards of men (Dixson, 2012).

One potential mechanism driving the evolution of dimorphic ornamentation is male-male competition. Under this mechanism, ornaments can be used to communicate male status and competitive ability to competitors/rivals at a distance, thereby averting costly/risky physical escalations (Rohwer, 1982). Examples of such badges of status are well documented among primates (e.g., Bergman et al., 2009; Gerald, 2001; Grueter et al., 2015; Järvistö et al., 2013; Koda et al., 2018; Møller, 1987; Setchell & Wickings, 2005; West & Packer, 2002; Zhou & Fuller, 2016). For instance, male mandrills feature rank-dependent red coloration on the face, rump and genitalia (Setchell & Wickings, 2005), and male black-and-white snub-nosed monkeys with reproductive monopoly over females possess brighter red lips than bachelor males (Grueter et al., 2015).

Another potential mechanism driving the evolution of dimorphic ornamentation is female choice. Here, the decorative traits convey information on the intrinsic quality of males that can be assessed by females during mate choice (Andersson, 1994; Barber, 1995; Dubuc et al., 2014; Jones & Ratterman, 2009; Koda et al., 2018; Kodric-Brown, 1985; Petrie et al., 1991; Setchell, 2005). For example, male rhesus macaques (*Macaca mulatta*) sporting darker red facial hues tend to attract more sexual attention from females compared to their counterparts with paler complexions (Dubuc et al., 2014). In humans, males exhibiting more masculine facial features are considered more appealing and tend to report having more sexual partners compared to men with less masculine facial characteristics (Rhodes et al., 2005).

The evolution of female ornaments in primates could also be the result of male mate choice. However, we refrain from exploring this scenario because their role of non-sex-specific colorful ornaments (i.e., ornaments expressed by both sexes) in male mate choice is not well understood. One study found that in Japanese macaques (*Macaca fuscata*) males do not take female coloration into account when making mating decisions (Rigaill & Garcia, 2021). However, in rhesus macaques, one study provided evidence that female skin redness was positively related to fecundity (Dubuc et al., 2014), and another study showed that females with redder faces mated more frequently and were courted for longer durations by males holding high ranks (Higham et al., 2021). Moreover, colorful ornaments can be an index of female reproductive state that males attend to (Dubuc et al., 2009). Overall, the lack of primates with more heavily ornamented females than males suggests the importance of female rather than male mate choice.

Across species, the extent of sexual dimorphism in ornaments has been shown to increase with group size (Grueter et al., 2015). One explanation for this association is that in large groups with numerous potentially unfamiliar individuals, it becomes far more practical and less energetically costly for individuals to advertise their characteristics via signals than through social recognition in direct interindividual interactions (Bergman & Sheehan, 2012; Grueter et al., 2015; Sheehan & Bergman, 2016). Although the social correlates of ornaments have been widely studied in a diversity of primate species (Bergman et al., 2009; Caro et al., 2021; Dixson et al., 2005; Grueter et al., 2015; Koda et al., 2018; Setchell & Wickings, 2005), these studies have focused almost exclusively on within-group signaling. However, given the dramatic interspecific variation not only in group size or structure, but also in the degree of exposure to, and interaction with, neighboring groups, at least some of these conspicuous signals may target in- as well as out-group members. Yet, little is known about the role of ornaments in extra-group signaling. Ornaments likely offer an opportunity for mutual visual assessment during confrontations. Males may signal their physical prowess and mate quality to an audience beyond their own group and in turn garner information about the characteristics of males in neighboring groups (e.g., individual resource holding potential, threat level). This information may also allow them to assess competition for their own dispersal opportunities. Likewise, females may derive valuable information from these signals about potential mating and dispersal opportunities. Morphological quality indicators may thus alleviate the challenge of individually recognizing numerous conspecifics. The only primate taxa for which some data on the beyond-group signaling potential of ornaments are available are those living in multilevel societies, where multiple core units stay in close proximity and go about their day-to-day activities in a seemingly coordinated manner (Grueter et al., 2020). In these societies, males likely signal to out-unit members with whom they are less familiar (Bergman et al., 2009; Grueter et al., 2015). Ornaments may also function to distinguish groups and not individuals per se, similar to emblems and appearance enhancers that often typify entire (ethnic) groups in humans and facilitate the distinction between in and out-group (Moffett, 2019; Sterelny, 2014).

To determine the extent to which the expression of ornamentation is linked to the social complexity at the between-group level in primates, we conducted comparative analyses relating the degree of sexual dimorphism in ornament expression (i.e., sexually selected status badges or quality indicators) to proxies of social complexity, such as home range overlap or intergroup encounter rates.

The rate of between-group encounters is often used to quantify the pressure exerted by outsiders (Ashton et al., 2020; Lemoine et al., 2020; Mosser & Packer, 2009). Home range overlap, which often results from multiple groups accessing spatially dispersed resources, could also be conceived as a quantitative measure of the prevalence/risk/probability of intrusions by neighboring groups and, by inference, as a proxy of inter-group competition (Crofoot, 2007; Grueter, 2013). That home range overlap reflects the frequency of mating competition is exemplified in colobine primates by greater sexual size dimorphism (a generally good indicator of male-male competition (Plavcan & van Schaik, 1997)) with more extensive home range overlap (Grueter & van Schaik, 2009). However, home range overlap could equally be suggestive of greater tolerance in some species (Ashton et al., 2020). To shed light on the usefulness of varying home range overlap as an index of mate competition between groups, we tested if the species-specific home range overlap was associated with the percentage of encounters that are agonistic. Irrespective of its role in competition, home range overlap can also be understood as a proxy of the extent of socio-spatial complexity at the intergroup level. In contrast to species with fixed territorial boundaries, primates with vastly overlapping home ranges are expected to regularly monitor the movements of groups in their vicinity and often encounter unfamiliar individuals that could pose serious threats to them (Grueter, 2015).

In sum, the aims of this study are to test the following hypotheses in a large sample of primate species: (1) Ornamentation is positively associated with both home range overlap and inter-group encounter rates (while controlling for variables previously shown to correlate with ornamentation, *viz*. group size and sexual size dimorphism; (Grueter et al., 2015)); (2) Species with greater home range overlap are characterized by a higher percentage of agonistic inter-group encounters.

## Materials and methods

### Data collection

**Figure 1.**
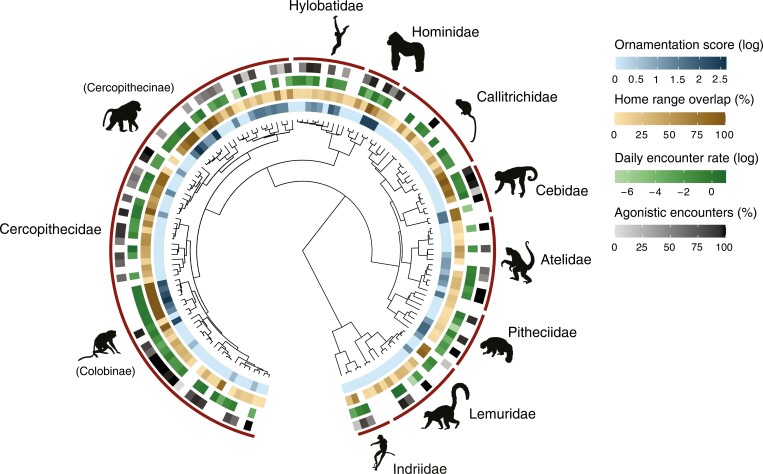
Consensus tree of 144 primate species with the main variables mapped to the tips. Note that the data are shown only for illustrative purposes of general patterns based on species means. The analyses themselves were conducted on up to five and seven data points per species for home range overlap and encounter rates, respectively. PhyloPic credit: A. Brouillet, K. Caspar, T.M. Keesey, M. Michaud, F. Sayol, S. Traver, S. Werning, and Dysalatornis (Terpsichores).

We collated information from the literature on home range overlap, inter-group encounter rates, the percentage of agonistic inter-group encounters, and sexual dimorphism in size and ornamentation for a large sample of primate species (both prosimian and anthropoid). Home range overlap was defined as the percentage of a group’s total range that is shared with neighboring groups. Encounter rate refers to the number of encounters between the focal group and neighboring groups per day. Agonistic encounters were defined as encounters that include any of the following behavioral elements: physical altercations, displays, avoidance, displacement, vigilance, and vocal exchanges. Data on home range overlap, inter-group encounter rates and the percentage of agonistic inter-group encounters were compiled predominantly from the peer-reviewed primary literature (to ensure accuracy) and not secondary sources. If the requisite data were available for multiple study sites of the same species, we included these raw data of all species. Data on home range overlap were available for 135 primate species, encounter rates for 99 species, and percentage of agonistic interactions for 71 species, across a total of 144 species with data for at least two of these variables (Figure 1). When collating the data, we disregarded heavily food provisioned populations (e.g., some Japanese macaque groups) and populations in non-native habitats (i.e., Cayo Santiago rhesus macaques). For a study to be included, it had to exceed a duration of 5 months of regular observations. Species with multilevel societies (multiple core units embedded within larger groups, (Grueter et al., 2020)) are characterized by complete range overlap among core units and were given a default between-unit encounter rate of 1 per day. The full dataset containing all variables is provided in the electronic Supporting Information (Supplementary Table 1).

Data on sexual dimorphism in ornamentation were sourced for 144 primate species. These are based on (Dixson et al., 2005; Grueter et al., 2015; Lüpold et al., 2019), but include slight modifications as well as species not previously sampled. We regarded as ornaments any fleshy swellings, contrasting coloration of exposed skin areas, and hairy traits such as beards, tufts or manes. Sexual dichromatism of the overall coat (e.g., many gibbon species) has not been definitively linked to sexual selection. The relatively inconspicuous pelage of male primates compared to females suggests that sexual selection might not be its primary driving force (Bartlett & Light, 2016; Bradley & Mundy, 2008). We therefore excluded these in the calculation for the metric for ornaments. We expressed the extent of sexual dimorphism in every discernible ornament of the trunk, limbs and head on a scale from 0 to 5: 0 = no difference in size and color between the sexes (e.g., both sexes have a crest of hair of the same size and color); 1 = a very small difference with the ornament slightly more developed in males than in females; 2 = a small but noticeable difference in males compared to females; 3 = moderate difference; 4 = large difference between the sexes; 5 = a very large difference (e.g., males possessing a prominent visual trait that is absent, or virtually so, in females). Supplementary Table 2 provides details of how the scoring system was applied to quantify degrees of dimorphism for each trait and gives examples of traits for each score. The ornamentation scores and descriptions of the individual traits for each species can be found in Supplementary Table 3. The sum of the scores for different trait categories (e.g., capes, tufts, beards or different fleshy or brightly colored traits) formed a species-specific value of sexual ornament dimorphism, which ranged between 0 and 13 across all species examined. All parts had the same weight in the final score.

We also analyzed the data with an alternative, more conservative system of scoring dimorphism in ornamentation (Lüpold et al., 2019). Here, we divided the body into three parts (head, trunk/limbs, and rump/anogenital area), which tend to vary independently in sexually dimorphic traits. For each body part, we scored fleshy swellings, skin patches, and hairy traits. Each of these trait categories was given a value of zero in the absence of any conspicuous sexual dimorphism, one for any visible sexual dimorphism in either structure or color or two for sexual dimorphism in both structure and color. Owing to the additive nature of these traits (e.g., *Mandrillus* and some *Papio* species have many strikingly dimorphic traits in multiple body parts, whereas other species differ primarily in one trait), we again summed across body regions all categorical scores for our analyses as in the first approach.

Finally, we also examined each of these three more specific ornamentation categories separately: Pelage dimorphism, genital coloration, and other traits like the nose of proboscis monkeys or lip warts in snub-nosed monkeys. This approach allowed us to determine if particular types of ornamentation were more likely to be considered by primates when encountering another social group, given a lack of clear empirical information on the relevant traits during such encounters.

### Analyses

We conducted all analyses using the *brms* package (Bürkner, 2017) for R v. 4.3.0. To account for phylogenetic non-independence in trait distributions, we incorporated a phylogenetic covariance matrix as a random effect into each model, and species identity as an additional random effect accounted for non-independence of data points within species. The underlying phylogenetic relationships derived from a set of 10,000 molecular species-level mammalian trees (Upham et al., 2019), from which we extracted all 317 primate species. Four species in our dataset (i.e., *Piliocolobus temminckii*, *P. epieni*, *P. tephrosceles*, and *Trachypithecus leucocephalus*) were not present in the phylogenetic tree. We inserted these into each tree sample based on and using branch lengths proportional to recent time-calibrated colobine phylogenies (Roos & Zinner, 2021). *Piliocolobus epieni*, which was not represented in either phylogeny, was inserted as a sister species to *P. tephrosceles* rather than *P. temminckii* in our pruned tree, as suggested by Ting (2008) based on both morphological and molecular similarities.

Due to considerable zero inflation in our (log-transformed) ornamentation scores, we fitted a hurdle model with a gamma distribution whenever total ornamentation was the response variable. Hurdle models test two processes simultaneously, a binary logit model predicting the probability of the outcome being zero (or not) and a zero-truncated regression model predicting the extent of the outcome (e.g., degree of ornamentation) across those values that are greater than zero (Cragg, 1971). For proportional response variables, such as the proportion of interactions that were agonistic, we performed beta regressions (Ferrari & Cribari-Neto, 2004) with a logit link in the mean model and a log link for the precision parameter *ϕ*. For proportional predictors, we arcsine square-root transformed these variables and then converted them to percentages by multiplying them by 180/π.

For all models, we ran 50 Markov Chain Monte Carlo (MCMC) chains across 15,000 iterations, with each chain using a different phylogeny randomly drawn from the 10,000 trees to incorporate phylogenetic uncertainty. Along each chain, we took 1,000 samples after 5,000 warm-up draws, with a thinning interval of 10 to reduce autocorrelation, before combining the samples across chains. We assessed the convergence, mixing and stability of the Bayesian sampling based on $\hat{R}$, which should be < 1.01 (Vehtari et al., 2021), the effective sample size that should exceed 1,000 (Bürkner, 2017), and visual inspection of the trace plots. Additionally, we assessed the prediction accuracy based on leave-one-out cross-validation using Pareto-smoothed importance sampling (in package *loo*; (Vehtari et al., 2017)) and predictive posterior checks (in package *bayesplot*; (Gabry & Mahr, 2021)). Finally, the posterior distributions from all 50 independent MCMC chains revealed high convergence, suggesting at best a minimal impact of the chosen phylogenetic tree on the final conclusions.

## Results

**Figure 2.**
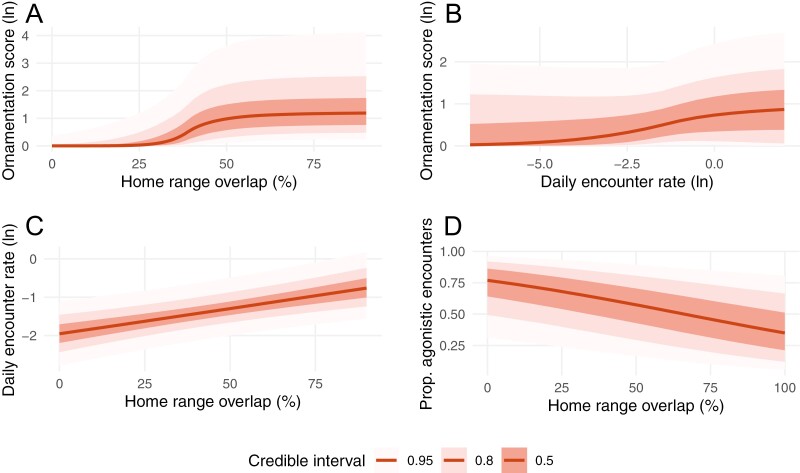
Predicted ornamentation scores in response to (A) percent home range overlap between groups and (B) daily encounter rate, as revealed by Bayesian gamma hurdle models. Shown are the combined effects of the hurdle and the mean parts of the model. (C) Daily encounter rate between groups in response to the percent overlap of their home ranges, as predicted by a Bayesian model with a skewed normal error distribution. (D) The proportion of agonistic encounters as predicted by home range overlap between groups in a Bayesian beta regression. The different colors indicate different credible intervals (50%, 80%, or 95%).

A hurdle model predicted enhancing ornamentation at increasing overlap between home ranges (Figure 2A; Supplementary Figure S1). This effect was indicated by a rapid shift from the absence to no absence of ornamentation between 25% and 40% home range overlap within the hurdle part (*β* = −0.34 [95% credible interval: −1.34, −0.04]) of the model, albeit not in the non-zero part (*β* = 0.0001 [−0.0004, 0.0007]). Since the main effect of increasing home range overlap was on the probability of being ornamented rather than its extent of ornamentation when present, the result using the more conservative estimates of ornamentation was essentially the same (hurdle part: *β* = −0.31 [−1.21, −0.03]; non-zero part: *β* = 0.0001 [−0.0004, 0.0007]). Finally, when considering the different types of ornamentation separately, we found strong support for home range overlap affecting the presence of skin or fleshy adornments (hurdle part: *β* = −0.57 [−2.32, −0.05]; non-zero part: *β* = 0.001 [−0.004, 0.007]), with weaker trends for pelage dimorphism (hurdle part: *β* = −0.20 [−0.86, 0.04]; non-zero part: *β* = 0.0002 [−0.0007, 0.0011]) and genital coloration (hurdle part: *β* = −0.12 [−0.47, 0.05]; non-zero part: *β* = 0.000 [−0.002, 0.002]).

Compared to home range overlap, there was no clear association of ornamentation with daily encounter rates between groups (gamma hurdle model on *N* = 173 from 99 species; hurdle part: *β* = −0.82 [−4.45, 1.65], non-zero part: *β* = 0.001 [−0.007, 0.009]; Figure 2B; Supplementary Figure S2). The same was true for the conservative ornamentation estimates (hurdle part: *β* = −0.87 [−4.56, 1.58], non-zero part: *β* = 0.001 [−0.008, 0.009]). Hence, ornamentation may have evolved in response to inter-group overlap in home ranges but less likely to the encounter rates, even though greater home range overlap between groups was associated with more frequent inter-group encounters (skewed-normal model due to right-tailed distribution: *N* = 122 from 90 species; *β* = 0.33 [0.10, 0.58]; Figure 2C; Supplementary Figure S3).

Given that ornamentation had previously been associated with both the degree of sexual size dimorphism and group size (Grueter et al., 2015), we repeated the above analyses, but this time controlling for these potentially confounding effects. The effect of home range overlap remained qualitatively the same (gamma hurdle model across *N* = 198 from 132 species; hurdle part: *β* = −8.25 [−32.50, −0.98], non-zero part: *β* = 0.002 [0.01, 0.02]), with a similarly positive effect of group size (*β* = 0.13 [0.04, 0.22]) but less so for sexual size dimorphism (*β* = 0.12 [−0.005, 0.24]; Supplementary Figure S4).

Finally, we examined if increased home range overlap or more daily encounters would result in more agonistic interactions. In beta regressions, we found that the percent home range overlap reduced the proportion of interactions that were agonistic (*N* = 78 from 62 species; *β* = −0.45 [−0.88, −0.04], *ϕ* = 0.84 [0.30, 1.42]; Figure 2D), whereas there was no evidence for an association between the daily encounter to do so (*N* = 101 from 61 species; *β* = −0.14 [−0.39, 0.011], *ϕ* = 0.27 [0.01, 0.55]; Supplementary Figure S5).

## Discussion

Across a large sample of primates, we showed that species with a greater proportion of space shared among neighboring groups exhibit more sexually dimorphic ornamentation. This finding suggests that investment in visually conspicuous markers may be adaptive in situations of greater exposure to outsiders. These markers thus seem to have evolved not only to facilitate interactions with potential adversaries and prospective mates within groups but also between groups.

Ornaments are particularly useful where individual recognition suffers or fails (Bergman, 2010; Bergman & Sheehan, 2012), such as in exceedingly large groups with higher levels of social anonymity (Grueter et al., 2015). The extent of individual recognition skills beyond the group level remains unclear, but it is plausible to hypothesize that extra-group social information is limited and that ornaments offer a rapid and approximate indicator for quality and status evaluation. The amplification of ornaments may thus be a substitute for mutual assessment not only within large groups but also and between groups. Ornamentation was not found to be clearly associated with encounter rate. The relationship between the two variables was positive and may imply higher demand for surveillance of potential rivals and thus greater need for ornaments. However, the relationship did not reach the strength of the one reported for home-range overlap and ornamentation. The lack of a strong causal link between this relationship requires more rigorous examination. It is possible that more frequent encounters facilitate recognition via social means and thus relax selection for phenotypic markers but this interpretation is difficult to reconcile with the strong positive effect of range overlap on ornamentation.

Our results also show that encounter rates do not predict the percentage of agonistic interactions. The variable “encounter rate” may not fully capture between-group competition for two reasons. First, if the information content of ornaments enables effective conflict resolution at a distance and obviates aggressive escalation, then the lack of a relationship between encounter rate and agonism is not surprising. Second, when between-group competition manifests as mutual avoidance, then low encounter rates may not necessarily imply low outsider pressure (Ashton et al., 2020).

There was good evidence that home range overlap is associated with agonistic interactions. The association between home range overlap and the percentage of agonistic encounters was negative, which suggests that species with greater home range overlap invest more in competitive signaling through ornaments, thereby reducing the need for direct aggression. Alternatively, the negative correlation between home range overlap and agonism implies higher levels of between-group competition in situations of more exclusive access to a home range (Seiler & Robbins, 2020), a potential sign of territoriality (Maher & Lott, 1995; Schoener, 1968; Willems et al., 2013); but see (Brown & Orians, 1970; Isbell et al., 2021). Species with higher home range overlap may show less aggression because the economic defensibility of the home range is reduced (Mitani & Rodman, 1979), which may render non-aggression in between-group contexts the more economic option (Pusey, 2022). This outcome is particularly expected in highly heterogeneous habitats with patchy/dispersed resource where multiple sympatric social units have the same spatial and dietary requirements (Grueter, 2022); see also (Macdonald & Johnson, 2015).

Regardless of its role in competition, home range overlap or lack of range exclusivity is a surrogate for the degree of socio-spatial complexity that primates experience (Grueter, 2015) and requires investments in signals that are effectively perceived from a distance. Facing exposure to spatial infringements on multiple fronts is also cognitively more taxing than having a fixed territory boundary (Grueter, 2015).

The analyses presented in this paper provide a conceptual basis for further studies and for extending them to other gregarious animals. Future studies could also explore alternative measures of between-group contact and outsider pressure that we did not test here, such as the number of fronts/neighbors or the frequency of extra-group matings (see (Ashton et al., 2020)). Another promising research direction would be to try to disentangle the effects of male-male competition and female choice in an inter-group context on the evolution of ornaments. Our study explored the role of ornaments as a means of navigating the beyond-group social sphere. It did not investigate how non-visual signals, such as vocalizations, olfaction, facial expressions and multimodal signals inform about the sender’s competitive ability or condition (Byrne & da Cunha, 2006; Dobson, 2009; Greene et al., 2016; Santana et al., 2014; Wright et al., 2021). These may either enhance or trade-off with visual signals.

In conclusion, we argue that a better understanding of the social evolution and adaptive function of individual-level traits such as ornaments requires going beyond the home group and acknowledging that group-living individuals are embedded in a broader social landscape that creates diverse selective pressures on them (Grueter et al., 2020; Morris-Drake et al., 2022).

## Supplementary material

Supplementary material is available online at *Evolution Letters*.

qrae045_suppl_Supplementary_Material

## Data Availability

All data and code are deposited on the Dryad Data Repository (doi: 10.5061/dryad.1g1jwsv5g).
