# Supplementary material for: The role of between-group signaling in the evolution of primate ornamentation
Source: Evol Lett. 2024 Aug 17;8(6):927–35. doi: 10.1093/evlett/qrae045 (PMC11637682; doi:10.1093/evlett/qrae045)
Supplement: qrae045_suppl_Supplementary_Material [file qrae045_suppl_supplementary_material.zip › SupplementaryInformation, CLEAN.docx]

Supplementary information to:

**The role of between-group signalling in the evolution of primate ornamentation**

**Cyril C. Grueter^1,2,3,4,*^, Stefan Lüpold^5^**

^1^ Department of Anatomy, Physiology and Human Biology, School of Human Sciences, The University of Western Australia, Perth, WA, Australia

^2^ International Centre of Biodiversity and Primate Conservation, Dali University, Dali, Yunnan, China

^3^ Centre for Evolutionary Biology, School of Biological Sciences, The University of Western Australia, Perth, WA, Australia

^4^ Centre of Excellence in Biodiversity and Natural Resource Management, University of Rwanda, Huye, Rwanda

^5^ Department of Evolutionary Biology and Environmental Studies, University of Zurich, Zurich, Switzerland

* Correspondence to: cyril.grueter@uwa.edu.au

**Table S1.** Variables used in the study.

| Species | Alternative taxonomy | Home range overlap [%] | Encounter rate (/d) | % agonistic encounters | Ornamentation score | Group size | Sexual dimorphism | REFs for range use variables | REFs for group size | REFs for sexual dimorphism |
| --- | --- | --- | --- | --- | --- | --- | --- | --- | --- | --- |
| *Alouatta belzebul* |  |  | 0.45 | 47.6 | 2 | 7.2 | 1.32 | Pinto et al. 2013 | Campbell et al. 2011 | Smith & Jungers 1997 |
| *Alouatta caraya* |  | 31 | 1.215 |  | 3 | 10.1 | 1.48 | Gennuso et al. 2018; Kowalewski & Garber 2015; Agostini et al. 2010; Bravo & Sallenave 2003 | Campbell et al. 2011 | Smith & Jungers 1997 |
| *Alouatta guariba* |  | 16.5 | 0.7 | 35 | 1 | 6.5 | 1.55 | Chiarello 1993, 1995; Agostini et al. 2010 | Campbell et al. 2011 | Smith & Jungers 1997 |
| *Alouatta palliata* |  | 32 | 0.26 | 100 | 2 | 15.1 | 1.34 | Willems-Guillén 2003; Hopkins 2011, 2013; Quintana-Morales et al. 2017 | Campbell et al. 2011 | Smith & Jungers 1997 |
| *Alouatta pigra* |  | 11 | 0.32 | 100 | 0 | 6.3 | 1.77 | Van Belle & Estrada 2020; Van Belle unpubl. | Campbell et al. 2011 | Smith & Jungers 1997 |
| *Alouatta seniculus* |  | 22.3 |  |  | 4 | 7.9 | 1.28 | Sekulic 1982a,b,c; Palma et al. 2011; Palacios & Rodriguez 2001; Gómez-Posada et al. 2007 | Campbell et al. 2011 | Smith & Jungers 1997 |
| *Ateles belzebuth* |  | 10 |  |  | 0 | 23.7 | 1.06 | Shimooka 2005 | Campbell et al. 2011 | Smith & Jungers 1997 |
| *Ateles chamek* | *Ateles belzebuth chamek* | 13 | 0.02 | 100 | 0 | 49.5 | 1.4 | Symington 1988 | Campbell et al. | Rowe 2016 |
| *Ateles geoffroyi* |  | 10 |  |  | 0 | 31.9 | 0.98 | Ramos-Fernández & Ayala-Orozco 2003 | Campbell et al. 2011 | Smith & Jungers 1997 |
| *Ateles paniscus* |  | 0 |  |  | 0 | 18 | 1.08 | Van Roosmalen 1985 | Campbell et al. 2011 | Smith & Jungers 1997 |
| *Brachyteles hypoxanthus* |  | 43.5 |  |  | 1 | 48 | 1.2 | Strier 1986, 1987; Dias & Strier 2003; Lima et al. 2019 | Campbell et al. 2011 | Rowe 2016 |
| *Callicebus discolor* | *Plecturocebus discolor* | 7 | 0.125 |  | 0 | 4 | 1.15 | Van Belle et al. 2021 | Rowe 2016 | Isler et al. 2008 |
| *Callicebus ornatus* | *Plecturocebus ornatus* | 5 | 0.85 | 80 | 0 | 4 | 1.01 | Mason 1968; Robinson 1979, 1981 | Rowe 2016 | Rowe 2016 |
| *Callicebus personatus* |  | 18 |  |  | 0 | 6 | 0.92 | Price & Piedade 2001 | Rowe 2016 | Smith & Jungers 1997 |
| *Callicebus lucifer* | *Cheracebus lucifer; Callicebus torquatus* | 10 | 0.00 |  | 0 | 4 |  | Kinzey & Robinson 1983 | Rowe 2016 |  |
| *Callimico goeldii* |  | 10 |  |  | 0 | 8 | 1.07 | Porter & Garber 2010 | Campbell et al. 2011 | Smith & Jungers 1997 |
| *Callithrix jacchus* |  | 56 | 1.38 |  | 0 | 9.5 | 0.98 | Lazaro-Perea 2011; Pontes & da Cruz 1995 | Campbell et al. 2011 | Smith & Jungers 1997 |
| *Callithrix aurita* |  | 15 |  |  | 0 | 7.5 | 1 | Ferrari et al. 1996 | Campbell et al. 2011 | Smith & Jungers 1997 |
| *Cebuella pygmaea* | *Callithrix pygmaea* | 0 |  |  | 0 | 5.5 | 0.9 | Soini 1982 | Campbell et al. 2011 | Smith & Jungers 1997 |
| *Cebus albifrons* |  | 80 | 0.26 |  | 0 | 19.8 | 1.39 | Matthews 2009; Terborgh 1983 | Campbell et al. 2011 | Smith & Jungers 1997 |
| *Cebus imitator* | *Cebus capucinus imitator* | 89 | 0.28 | 100 | 0 | 17 | 1.45 | Schoof & Jack 2013; Perry 1996; Crofooot 2008 | Rowe 2016 | Smith & Jungers 1997 |
| *Cebus olivaceus* |  | 100 | 0.46 | 80 | 0 | 21 | 1.31 | Robinson 1986, 1988 | Campbell et al. 2011 | Smith & Jungers 1997 |
| *Cercocebus galeritus* |  | 90 |  | 32.7 | 0 | 28.5 | 1.83 | Kinnaird 1992 | Campbell et al. 2011 | Smith & Jungers 1997 |
| *Cercopithecus ascanius* |  | 18 | 0.26 | 49 | 0 | 28.4 | 1.27 | Brown 2013, Brown unpubl. | Campbell et al. 2011 | Smith & Jungers 1997 |
| *Cercopithecus campbelli* |  | 55 | 0.03 | 67 | 0 | 9.3 | 1.67 | Buzzard & Eckardt 2007 | Campbell et al. 2011 | Smith & Jungers 1997 |
| *Cercopithecus cephus* |  | 8 |  |  | 0 | 6 | 1.49 | Willems et al. 2013 | Campbell et al. 2011 | Smith & Jungers 1997 |
| *Cercopithecus diana* |  | 66 | 0.36 | 35 | 3 | 24.1 | 1.33 | Buzzard & Eckardt 2007 | Campbell et al. 2011 | Smith & Jungers 1997 |
| *Cercopithecus mitis* |  | 27.75 | 3.835 | 70 | 2 | 23.9 | 1.87 | Lawes & Henzi 1995; Payne et al. 2003; Roth et al. 2016; Butynski 1990 | Campbell et al. 2011 | Smith & Jungers 1997 |
| *Cercopithecus neglectus* |  | 10 |  | 10 | 3 | 9.2 | 1.78 | Gautier-Hion & Gautier 1978; Wahome et al. 1993 | Campbell et al. 2011 | Smith & Jungers 1997 |
| *Cercopithecus nictitans* |  | 0 | 0 |  | 0 | 12 | 1.57 | Buzzard & Eckardt 2007 | Campbell et al. 2011 | Smith & Jungers 1997 |
| *Cercopithecus petaurista* |  | 64 | 0.13 | 31 | 3 | 10.7 | 1.52 | Buzzard & Eckardt 2007 | Campbell et al. 2011 | Smith & Jungers 1997 |
| *Chiropotes sagulatus* |  |  | 0.05 |  | 2 | 25 | 1.15 | Shaffer 2012 | Campbell et al. 2011 | Rowe 2016 |
| *Chlorocebus pygerythryus* | *Cercopithecus aethiops* | 33.18 | 0.36 | 67.5 | 4 | 45 | 1.57 | Struhsaker 1967a,b; Cheney 1981, 1987; Isbell et al. 1990, 2021; Arseneau-Robar et al. 2016 | Rowe 2016 | Gordon 2006 |
| *Chlorocebus sabaeus* | *Cercopithecus sabaeus; Cercopithecus aethiops* | 9.6 | 0.25 |  | 3 | 26 | 1.61 | Harrison 1983 | Mittermeier 2013 | Plavcan & Ruff 2008 |
| *Chlorocebus tantalus* | *Cercopithecus aethiops* | 12 |  | 10 | 4 | 44 | 1.35 | Kavanagh 1981 | Rowe 2016 | Rowe 2016 |
| *Colobus angolensis* |  | 100 | 1 |  | 2 | 81 | 1.28 | Miller et al. 2020; Stead & Teichroeb 2019; Teichroeb et al. 2022 | Campbell et al. 2011 | Smith & Jungers 1997 |
| *Colobus guereza* |  | 61 | 1.01 | 77.7 | 0 | 9 | 1.47 | Harris 2006; Oates 1977a,c; Chapman & Pavelka 2005; von Hippel 1996; Fashing 2001a,c | Campbell et al. 2011 | Smith & Jungers 1997 |
| *Colobus polykomos* |  | 12.5 | 0.12 | 77.5 | 0 | 13.6 | 1.19 | Oates 1994; Dasilva 1989 | Campbell et al. 2011 | Smith & Jungers 1997 |
| *Colobus satanas* |  | 65 | 0.16 |  | 0 | 13.7 | 1.4 | Fleury & Gautier-Hion 1999; McKey & Waterman 1982; McKey 1978 | Campbell et al. 2011 | Smith & Jungers 1997 |
| *Colobus vellerosus* |  | 33 | 1.15 | 85.2 | 0 | 15.2 | 1.23 | Sicotte & Macintosh 2004; Saj & Sicotte 2007; Teichroeb & Sicotte 2018 | Campbell et al. 2011 | Smith & Jungers 1997 |
| *Eulemur collaris* |  |  | 0.36 |  | 2.5 | 5 | 0.93 | Campera et al. 2014 | Campbell et al. 2011 | Rowe 2016 |
| *Eulemur fulvus* |  | 80 |  | 10 | 0 | 11.5 | 1.05 | Pollock 1979; Sato 2013 | Rowe 2016 | Rowe 2016 |
| *Eulemur flavifrons* |  | 31 | 0.00 |  | 0 | 8 | 1.07 | Volampeno et al. 2011 | Rowe 2016 | Smith & Jungers 1997 |
| *Eulemur macaco* |  | 33 |  |  | 0 | 10 | 0.94 | Bayart & Simmon 2005 | Campbell et al. 2011 | Smith & Jungers 1997 |
| *Eulemur mongoz* |  | 31 |  |  | 0 | 5.5 | 1.01 | Curtis & Zaramody 1998 | Campbell et al. 2011 | Smith & Jungers 1997 |
| *Eulemur rubriventer* |  | 10 | 0.12 | 100 | 0 | 3 | 1.02 | Overdorff & Tecot 2006 | Campbell et al. 2011 | Smith & Jungers 1997 |
| *Eulemur rufus* |  | 90 | 0.52 |  | 0 | 11 | 0.97 | Gould & Overdorff 2002; Ostner et al. 2008 | Rowe 2016 | Smith & Jungers 1997 |
| *Erythrocebus patas* |  | 10 |  |  | 4 | 30 | 1.91 | Enstam et al. 2002 | Campbell et al. 2011 | Smith & Jungers 1997 |
| *Gorilla beringei* |  | 61.5 | 0.06 | 87.5 | 10 | 12.2 | 1.67 | Sicotte 1993; Mirville et al. 2018; Caillaud et al. 2014; Robbins & Sawyer 2007; Seiler et al. 2017 | Rowe 2016 | Smith & Jungers 1997 |
| *Gorilla gorilla* |  | 27 | 0.87 | 35.9 | 10 | 12.8 | 2.38 | Cooksey et al. 2020; Doran-Sheehy et al. 2004; Bermejo 2004; Forcina et al. 2019; Greenway 2015; Parnell 2002; Magliocca & Gautier-Hion 2004; Caillaud et al. 2008; Levero 2005; Gatti et al. 2004 | Rowe 2016 | Smith & Jungers 1997 |
| *Hapalemur griseus alaotrensis* | *Hapalemur alaotrensis* | 5 | 1.44 | 56 | 0 | 4.4 | 1.09 | Nievergelt et al. 1998 | Rowe 2016 | Smith & Jungers 1997 |
| *Homo sapiens* |  | 100 | 1.00 |  | 10 | 490 | 1.16 | Willems et al. 2013; Grueter 2015 | Layton et al. 2012 | Smith & Jungers 1997 |
| *Hoolock hoolock* |  | 9 |  |  | 0 | 3 | 1 | Willems et al. 2013 | Rowe 2016 | Smith & Jungers 1997 |
| *Hylobates agilis* |  | 24 | 0.45 | 80 | 3 | 7 | 1.01 | Gittins 1980 | Rowe 2016 | Smith & Jungers 1997 |
| *Hylobates albibaris* |  | 16.5 |  |  | 2 | 4.4 | 0.85 | Cheyne et al. 2008, 2019 | Rowe 2016 | Rowe 2016 |
| *Hylobates klossi* |  | 10 | 0.4 |  | 0 | 4.3 | 0.96 | Tenaza 1975 | Rowe 2016 | Smith & Jungers 1997 |
| *Hylobates lar* |  | 42 | 0.53 | 58 | 0 | 4.1 | 1.1 | Bartlett 2003; Reichard & Sommer 1997; Suwanvecho & Brockelman 2012; Ellefson 1974; Chivers & Raemaekers 1980 | Rowe 2016 | Smith & Jungers 1997 |
| *Hylobates moloch* |  | 8 | 0.475 | 40 | 0 | 3.5 | 1 | Kappeler 1984; Yi et al. 2020 | Rowe 2016 | Smith & Jungers 1997 |
| *Hylobates muelleri* |  | 11 | 0.06 | 86 | 2 | 3.2 | 1.07 | Mitani 1985 | Rowe 2016 | Smith & Jungers 1997 |
| *Hylobates pileatus* |  | 18.5 | 0.17 |  | 2.5 | 4 | 1.01 | Suwanvecho & Brockelman 2012; Brockelman & Srikosamatara 1984 | Rowe 2016 | Smith & Jungers 1997 |
| *Indri indri* |  | 22 | 0.04 | 10 | 0 | 2 | 0.85 | Pollock 1975, 1979; Bonadonna et al. 2017; Powzyck 1997 | Rowe 2016 | Plavcan & Ruff 2008 |
| *Lagothrix lagothricha* | *Lagothrix poeppigii* | 53 | 0.19 | 63 | 0 | 31.8 | 1.04 | Di Fiore 1997; Di Fiore 2003; Defler 1996; Ellis & Di Fiore 2019 | Campbell et al. 2011 | Smith & Jungers 1997 |
| *Lagothrix flavicauda* | *Oreonax flavicauda* | 1.4 |  |  | 2 | 11 | 1.6 | Shanee 2014 | Campbell et al. 2011 | Rowe 2016 |
| *Lemur catta* |  | 42 | 2.09 | 87 | 0 | 13.8 | 1 | Nakamichi & Koyama 1997; Jolly et al. 1993; Gould & Overdorff 2002 | Campbell et al. 2011 | Smith & Jungers 1997 |
| *Leontopithecus chrysomelas* |  | 9 | 0.26 | 100 | 0 | 6.5 | 1.16 | Rylands 1989; Raboy & Dietz 2004 | Campbell et al. 2011 | Smith & Jungers 1997 |
| *Leontopithecus rosalia* |  | 61 | 0.54 |  | 0 | 6.5 | 1.04 | Peres 1989 | Campbell et al. 2011 | Smith & Jungers 1997 |
| *Lophocebus albigena* | *Lophocebus ugandae; Cercocebus albigena* | 48 | 0.09 | 43 | 0 | 16.1 | 1.37 | Waser 1976; Struhsaker & Leland 1979; Brown 2013 | Campbell et al. | Smith & Jungers 1997 |
| *Macaca fascicularis* |  | 67 | 0.45 | 46 | 3 | 27.5 | 1.49 | Pal et al. 2018; Willems et al. 2013 | deCasien et al. 2017 | Smith & Jungers 1997 |
| *Macaca fuscata* |  | 59 | 0.32 | 55.46 | 0 | 64.7 | 1.37 | Hanya et al. 2008; Sugiura et al. 2000; Maruhashi et al. 1998; Majolo et al. 2005 | deCasien et al. 2017 | Smith & Jungers 1997 |
| *Macaca leonina* |  |  | 0.11 | 100 | 2 | 23.3 | 1.57 | Albert et al. 2011; Choudhury 2008 | Rowe 2016 | Smith & Jungers 1997 |
| *Macaca maura* |  |  | 0.42 |  | 0 | 30 | 1.41 | Okamoto & Matsumura 2002 | Rowe 2016 | Rowe 2016 |
| *Macaca mulatta* |  | 70 | 0.44 | 37 | 0 | 39.2 | 1.25 | Lindburg 1971, 1977 | Rowe 2016 | Smith & Jungers 1997 |
| *Macaca nemestrina* |  | 88 |  |  | 0 | 31.6 | 1.72 | Oi 1990; Bernstein 1967 | deCasien et al. 2017 | Smith & Jungers 1997 |
| *Macaca nigra* |  | 48 |  |  | 0 | 47 | 1.81 | O’Brien & Kinnaird 1997 | deCasien et al. 2017 | Smith & Jungers 1997 |
| *Macaca radiata* |  |  | 1.085 | 30.85 | 0 | 28.9 | 1.73 | Cooper et al. 2004 | deCasien et al. 2017 | Smith & Jungers 1997 |
| *Macaca silenus* |  | 10 | 0.1 | 85.7 | 0 | 19 | 1.46 | Santhosh et al. 2015; Kumara et al. 2014 | deCasien et al. 2017 | Smith & Jungers 1997 |
| *Macaca sylvanus* |  | 57.5 | 0.44 | 28 | 0 | 21.9 | 1.45 | Deag 1973; Mehlman & Parkhill 1988; Majolo et al. 2013 | deCasien et al. 2017 | Smith & Jungers 1997 |
| *Macaca tonkeana* |  | 10 |  |  | 0 | 10 | 1.66 | Pombo et al. 2004 | Rowe 2016 | Smith & Jungers 1997 |
| *Mico intermedius* | *Callithrix humeralifer* | 22 |  | 80 | 0 | 12 | 0.9 | Rylands 1986 | Campbell et al. 2011 | Mittermeier 2013 |
| *Nasalis larvatus* |  | 94.33 | 0.68 |  | 9 | 30 | 2.09 | Boonratana 1993, 2000; Bennett & Sebastian 1988; Yeager 1989, 1990, 1991 | Grueter 2009 | Grueter & van Schaik 2009 |
| *Nomascus hainanus* |  | 11.5 |  |  | 0 | 6 | 1.01 | Bryant et al. 2017 | Li et al. 2022 | Rowe 2016 |
| *Pan paniscus* |  | 52.2 | 0.69 | 48.5 | 0 | 30.5 | 1.36 | Idani 1990; Hashimoto et al. 1998; Sakamaki et al. 2018; Hohmann & Fruth 2002; Samuni et al. 2020; Moscovice et al. 2022 | Campbell et al. 2011 | Smith & Jungers 1997 |
| *Pan troglodytes* |  | 13.5 | 0.02 | 86 | 0 | 49.3 | 1.23 | Williams et al. 2004; Goodall 1979; Boesch et al. 2008; Herbinger et al. 2001; Watts & Mitani 2001; Martínez-Íñigo et al. 2021 | Campbell et al. 2011 | Smith & Jungers 1997 |
| *Papio anubis* |  | 80 | 0.384 |  | 4 | 49.3 | 1.89 | DeVore & Hall 1965; Harding 1976 | Campbell et al. 2011 | Smith & Jungers 1997 |
| *Papio cynocephalus* |  | 25 |  |  | 2 | 55.3 | 1.77 | Shopland 1982 | Campbell et al. 2011 | Smith & Jungers 1997 |
| *Papio hamadryas* |  | 100 | 1 |  | 13 | 85.6 | 1.84 | Kummer 1968 | Campbell et al. 2011 | Smith & Jungers 1997 |
| *Papio papio* |  | 100 | 1 |  | 6 | 60 | 1.92 | Fischer et al. 2017 | Patzelt et al. 2014 | Rowe 2016 |
| *Papio ursinus* |  | 43 | 0.36 | 31.9 | 2 | 48.7 | 2.01 | Cowlishaw 1995; Byrne et al. 1987; Anderson 1981; Slater et al. 2018; Kitchen et al. 2004; Stoltz & Saayman 1970; Cheney & Seyfarth 1977; Hamilton et al. 1975; Hoffman & O’Riain 2012 | Campbell et al. 2011 | Smith & Jungers 1997 |
| *Piliocolobus badius* |  | 6 |  |  | 0 | 42.5 | 1.02 | Kortjens 2001 | Rowe 2016 | Smith & Jungers 1997 |
| *Piliocolobus epieni* | *Procolobus badius* | 42 | 0.10 | 67 | 0 | 46 |  | Were 2000 | Campbell et al. 2011 |  |
| *Piliocolobus rufomitratus* | *Procolobus badius* | 36 | 0.09 | 50 | 0 | 11.2 | 1.34 | Marsh 1979 | Campbell et al. 2011 | Smith & Jungers 1997 |
| *Piliocolobus temminckii* | *Piliocolobus badius temminckii; Procolobus badius* | 60 |  | 40 | 0 | 26 | 1.25 | Starin 1991 | Rowe 2016 | Rowe 2016 |
| *Piliocolobus tephrosceles* | *Procolobus badius* | 45 | 2.244 |  | 0 | 63.5 | 1.34 | Struhsaker 2010; Chapman & Pavelka 2005 | Campbell et al. 2011 | Rowe 2016 |
| *Piliocolobus kirkii* |  | 48 |  |  | 0 | 34.3 | 1.06 | Siex 2003 | Campbell et al. 2011 | Smith & Jungers 1997 |
| *Pithecia aequatorialis* |  | 5 | 0.02 |  | 0 | 4 |  | Van Belle et al. 2018 | Campbell et al. 2011 |  |
| *Pithecia irrorata* |  | 15.8 | 0.45 | 80 | 2 | 3.1 | 1.09 | Palminteri et al. 2012, 2016; Palminteri & Peres 2012 | Campbell et al. 2011 | Smith & Jungers 1997 |
| *Pithecia pithecia* |  | 8 | 0.14 | 100 | 0 | 4.6 | 1.23 | Thompson et al. 2012 | Campbell et al. 2011 | Smith & Jungers 1997 |
| *Presbytis comata* |  | 9 | 0.42 | 100 | 0 | 6.7 | 1.01 | Ruhiyat 1983 | Grueter & van Schaik 2010 | Grueter and van Schaik 2009 |
| *Presbytis femoralis* |  | 31 | 0.66 | 100 | 0 | 9 | 1.01 | Megantara 1989 | Nijman 2022 | Isler et al. 2008 |
| *Presbytis potenziani* |  | 34 | 0.132 | 100 | 1 | 3.8 | 1.02 | Sangchantr 2004; Fuentes 1996 | Grueter & van Schaik 2010 | Grueter & van Schaik 2009 |
| *Presbytis rubicunda* |  | 27.75 | 0.2048 | 89.67 | 0 | 6.4 | 1.05 | Salafsky 1988; Davies 1984; van Schaik et al. 1992; Supriatna et al. 1986; Ehlers Smith 2014 | Grueter & van Schaik 2010 | Grueter & van Schaik 2009 |
| *Presbytis sabana* | *Presbytis hosei* | 10.2 | 0.043 | 50 | 0 | 7.5 | 1.11 | Mitchell 1994 | Grueter & van Schaik 2010 | Grueter and van Schaik 2009 |
| *Presbytis siamensis* |  | 40.67 | 0.474 | 100 | 0 | 14.2 | 1.02 | Bennett 1983, 1986; Curtin 1980; Johns 1983 | Nijman 2022 | Grueter and van Schaik 2009 |
| *Presbytis thomasi* |  | 25.5 | 0.20335 | 66 | 0 | 8.9 | 1.01 | Gurmaya 1986; Assink & van Dijk 1990; van Schaik et al. 1992; Steenbeek 1999a,b; Wich & Sterck 2007 | Grueter & van Schaik 2010 | Smith & Jungers 1997 |
| *Procolobus verus* |  | 14 |  | 50 | 0 | 7.8 | 1.12 | Korstjens & Noe 2004; Korstjens 2001 | Campbell et al. 2011 | Smith & Jungers 1997 |
| *Propithecus diadema* |  | 5 |  |  | 0 | 4.8 | 0.95 | Willems et al. 2013; Powzyk 1997 | Campbell et al. 2011 | Smith & Jungers 1997 |
| *Propithecus edwardsi* |  | 10 |  |  | 0 | 5.3 | 0.96 | Gerber et al. 2012 | Campbell et al. 2011 | Smith & Jungers 1997 |
| *Propithecus coronatus* |  |  | 0.84 | 37 | 0 | 5 | 1 | Ramanamisata et al. 2014 | Campbell et al. 2011 | Pichon & Simmen 2015 |
| *Propithecus verreauxi* |  | 65.25 | 0.59 | 67.5 | 0 | 6 | 1.1 | Koch 2015; Lewis et al. 2020; Benadi et al. 2008; Jolly 1966; Richard 1977 | Campbell et al. 2011 | Smith & Jungers 1997 |
| *Pygathrix nigripes* |  | 100 | 1 |  | 5 | 18.5 | 1.3 | Hoang 2007 | Grueter et al. 2022 | Plavcan & Ruff 2008 |
| *Pygathrix nemaeus* |  | 100 | 1 |  | 3 | 26.7 | 1.3 | Ulibarri 2013 | Grueter et al. 2022 | Smith & Jungers 1997 |
| *Rhinopithecus avunculus* |  | 100 | 1 |  | 5 | 80 | 1.75 | Boonratana & Le 1994 | Grueter & van Schaik 2010 | Grueter & van Schaik 2009 |
| *Rhinopithecus bieti* |  | 100 | 1 |  | 8 | 210 | 1.68 | Xiang et al. 2013; Grueter et al. 2017 | Grueter & van Schaik 2010 | Grueter & van Schaik 2009 |
| *Rhinopithecus brelichi* |  | 100 | 1 |  | 12 | 400 | 1.86 | Bleisch et al. 1993; Nie et al. 2009 | Grueter & van Schaik 2010 | Grueter & van Schaik 2009 |
| *Rhinopithecus roxellana* |  | 100 | 1 |  | 8 | 215 | 1.74 | Ren et al. 1998; Tan et al. 2007; Zhang et al. 2006; Qi et al. 2014 | Grueter & van Schaik 2010 | Grueter & van Schaik 2009 |
| *Saguinus fuscicollis* | *Leontocebus fuscicollis* | 43 | 0.14 |  | 0 | 6 | 0.96 | Terborgh 1983; Peres 2000 | Campbell et al. 2011 | Smith & Jungers 1997 |
| *Saguinus niger* |  | 21.7 |  |  | 0 | 5 | 1.01 | Oliveira & Ferrari 2008 | Rowe 2016 | Rowe 2016 |
| *Saguinus imperator* |  | 10 | 0.14 |  | 0 | 4.8 | 1 | Terborgh 1983 | Rowe 2016 | Smith & Jungers 1997 |
| *Saguinus midas* |  | 21 | 0.79 |  | 0 | 7.5 | 0.9 | Day & Elwood 1999 | Rowe 2016 | Smith & Jungers 1997 |
| *Saguinus oedipus* |  | 13 |  |  | 0 | 6 | 1.03 | Dawson 1979 | Campbell et al. 2011 | Smith & Jungers 1997 |
| *Saguinus mystax* |  | 42.25 | 0.93 |  | 0 | 7 | 0.95 | Garber et al. 1993; Peres 1992; Stojan-Dolar & Heymann 2010 | Campbell et al. 2011 | Smith & Jungers 1997 |
| *Saguinus nigricollis* | *Leontocebus nigricollis* | 83 |  |  | 0 | 8 | 0.97 | de la Torre et al. 1995 | Rowe 2016 | Smith & Jungers 1997 |
| *Saimiri oerstedii* |  | 80 | 0.004 |  | 4 | 50 | 1.32 | Boinski 1987; Mitchell et al. 1991 | Campbell et al. 2011 | Smith & Jungers 1997 |
| *Saimiri sciureus* |  | 80 |  |  | 4 | 23 | 1.18 | Mitchell et al. 1991 | Campbell et al. | Smith & Jungers 1997 |
| *Sapajus apella macrocephalus* | *Sapajus macrocephalus; Cebus apella* | 40 | 0.865 | 10 | 0 | 16 | 1.6 | Defler 1982; Terborgh 1983 | Rowe 2016 | Rowe 2016 |
| *Sapajus nigritus* | *Cebus apella* |  |  | 100 | 0 | 14.5 | 1.47 | Di Bitetti 2001; Van Belle & Scarry 2015 | Rowe 2016 | Isler et al 2008 |
| *Semnopithecus entellus* |  | 25 | 0.76 | 44.33 | 0 | 21.2 | 1.31 | Starin 1978; Newton 1992; Newton & Dunbar 1994; Bennett & Davies 1994; Sugiyama 1967; Yoshina 1968; Hrdy 1977; Jay 1965; Vogel 1975 | Grueter & van Schaik 2010 | Smith & Jungers 1997 |
| *Semnopithecus johnii* | *Trachypithecus johnii* | 10 |  | 74 | 0 | 7 | 1.2 | Poirier 1968, 1970; Bennett & Davies 1994 | Grueter & van Schaik 2010 | Grueter & van Schaik 2009 |
| *Semnopithecus priam* | *Semnopithecus entellus* | 10 | 1.20 | 84 | 0 | 31.1 | 1.65 | Ripley 1967 | Grueter & van Schaik 2010 | Grueter & van Schaik 2009 |
| *Semnopithecus schistaceus* | *Semnopithecus entellus* |  | 0.06 |  | 1 | 35.2 | 1.3 | Curtin 1975; Sugiyama 1976; Bishop 1975, 1979 | Grueter & van Schaik 2010 | Smith & Jungers 1997 |
| *Semnopithecus vetulus* | *Trachypithecus vetulus* | 4 |  |  | 0 | 8.9 | 1.21 | Moore et al. 2010 | Grueter & van Schaik 2010 | Grueter & van Schaik 2009 |
| *Simias concolor* |  | 7.25 | 0.25 | 91.5 | 0 | 5.4 | 1.23 | Watanabe 1981; Tenaza & Fuentes 1995; Erb 2012 | Grueter et al. 2022 | Grueter & van Schaik 2009 |
| *Symphalangus syndactylus* |  | 30 | 0.14 | 28 | 3.5 | 4 | 1.11 | Chivers 1974 | Rowe 2016 | Smith & Jungers 1997 |
| *Theropithecus gelada* |  | 100 | 1 |  | 13 | 156.2 | 1.62 | Dunbar & Dunbar 1975 | Campbell et al. 2011 | Smith & Jungers 1997 |
| *Trachypithecus auratus* |  | 23 | 0.64 | 65.7 | 0 | 14 | 1 | Vogt 2003; Kool 1989 | Grueter & van Schaik 2010 | Grueter et al. 2015 |
| *Trachypithecus crepusculus* | *Trachypithecus phayrei* | 4.3 |  |  | 0 | 17 | 1.08 | Gibson & Koenig 2012; Fan PF, pers. comm. Aug. 2021 | Rowe 2016 | Rowe 2016 |
| *Trachypithecus cristatus* |  | 2 | 0.306 |  | 0 | 20.3 | 1.15 | Bernstein 1968 | Rowe 2016 | Smith & Jungers 1997 |
| *Trachypithecus geei* |  |  |  | 0 | 0 | 10.7 | 1.15 | Mukherjee & Saha 1974 | Grueter & van Schaik 2010 | Grueter and van Schaik 2009 |
| *Trachypithecus leucocephalus* |  | 16 |  | 48 | 0 | 10.3 | 1.12 | Li & Rogers 2005 | Grueter & van Schaik 2010 | Grueter and van Schaik 2009 |
| *Trachypithecus obscurus* |  | 3 | 0.0336 | 100 | 0 | 17 | 1.19 | Curtin 1980 | Grueter & van Schaik 2010 | Grueter and van Schaik 2009 |
| *Trachypithecus pileatus* |  | 84 | 0.96 |  | 0 | 8.6 | 1.22 | Stanford 1991 a,b | Grueter & van Schaik 2010 | Smith & Jungers 1997 |
| *Trachypithecus poliocephalus* |  | 17 |  |  | 0 | 8 | 1.19 | Hendershott et al. 2018 | Schneider et al. 2010 | Grueter et al. 2015 |
| *Varecia rubra* | *Varecia variegata rubra* | 9 |  |  | 0 | 24.5 | 1.02 | Rigamonti 1993 | Campbell et al. 2011 | Rowe 2016 |
| *Varecia variegata* |  | 41.5 |  |  | 0 | 8 | 1.03 | Holmes et al. 2019 | Campbell et al. 2011 | Smith & Jungers 1997 |

**References**

Agostini, I., Holzmann, I., & Di Bitetti, M. S. (2010). Ranging patterns of two syntopic howler monkey species (*Alouatta guariba* and *A. caraya*) in Northeastern Argentina. *International Journal of Primatology, 31*(3), 363-381.

Alba-Mejia, L., Caillaud, D., Montenegro, O. L., Sánchez-Palomino, P., & Crofoot, M. C. (2013). Spatiotemporal interactions among three neighboring groups of free-ranging white-footed tamarins (*Saguinus leucopus*) in Colombia. *International Journal of Primatology, 34*(6), 1281-1297.

Albert, A., Savini, T., & Huynen, M. C. (2011). Sleeping site selection and presleep behavior in wild pigtailed macaques. *American Journal of Primatology, 73*(12), 1222-1230.

Anderson, C. M. (1981). Intertroop relations of chacma baboon (*Papio ursinus*). *International Journal of Primatology, 2*(4), 285-310.

Arseneau-Robar, T. J. M., Taucher, A. L., Müller, E., van Schaik, C., Bshary, R., & Willems, E. P. (2016). Female monkeys use both the carrot and the stick to promote male participation in intergroup fights. *Proceedings of the Royal Society B: Biological Sciences, 283*(1843), 20161817.

Assink, P., & van Dijk, I. (1990). *Social organization, ranging and density of Presbytis thomasi at Ketambe (Sumatra), and a comparison with other Presbytis species at several South-east Asian locations.* (Doctoral thesis). University of Utrecht.

Bartlett, T. Q. (2003). Intragroup and intergroup social interactions in white-handed gibbons. *International Journal of Primatology, 24*, 239-259.

Bayart, F., & Simmen, B. (2005). Demography, range use, and behavior in black lemurs (*Eulemur macaco macaco*) at Ampasikely, northwest Madagascar. *American Journal of Primatology, 67*(3), 299-312.

Benadi, G., Fichtel, C., & Kappeler, P. (2008). Intergroup relations and home range use in Verreaux's sifaka (*Propithecus verreauxi*). *American Journal of Primatology, 70*(10), 956-965.

Bennett, E. (1983). *The Banded Langur: Ecology of a Colobine in West Malaysian Rain-Forest.* (Ph.D. thesis). Sidney Sussex College, Cambridge.

Bennett, E. L. (1986). Environmental correlates of ranging behaviour in the banded langur, *Presbytis melalophos*. *Folia Primatologica, 47*, 26-38.

Bennett, E. L., & Davies, A. G. (1994). The ecology of Asian colobines. In A. G. Davies & J. F. Oates (Eds.), *Colobine Monkeys: Their Ecology, Behaviour and Evolution* (pp. 129-171). Cambridge: Cambridge University Press.

Bennett, E. L., & Sebastian, A. C. (1988). Social organization and ecology of proboscis monkeys (*Nasalis larvatus*) in mixed coastal forest in Sarawak. *International Journal of Primatology, 9*, 233-255.

Bermejo, M. (2004). Home-range use and intergroup encounters in western gorillas (*Gorilla g. gorilla*) at Lossi Forest, North Congo. *American Journal of Primatology*, *64*, 223-232.

Bernstein, I. S. (1967). A field study of the pigtail monkey (*Macaca nemestrina*). *Primates, 8*(3), 217-228.

Bernstein, I. S. (1968). The lutong of Kuala Selangor. *Behaviour, 32*(1-3), 1-16.

Bishop, N. (1975 ). *Social Behavior of Langur Monkeys (Presbytis entellus) in a High Altitude Environment.* (Ph.D.). University of California Berkeley.

Bishop, N. (1979). Himalayan langurs: temperate colobines *Journal of Human Evolution, 8*, 251-281

Bleisch, W. V., Cheng, A. S., Ren, X. D., & Xie, J. H. (1993). Preliminary results from a field study of wild Guizhou snub-nosed monkeys (*Rhinopithecus brelichi*). *Folia Primatologica, 60*, 72-82.

Bocian, C. (1997). *Niche Separation of Black-and-White Colobus monkeys (Colobus angolensis and C. guereza) in the Ituri Forest.* (Ph.D. thesis). City University of New York, New York.

Boesch, C., Crockford, C., Herbinger, I., Wittig, R., Moebius, Y., & Normand, E. (2008). Intergroup conflicts among chimpanzees in Tai National Park: lethal violence and the female perspective. *American Journal of Primatology, 70*, 519-532.

Boinski, S. (1987). Habitat use by squirrel monkeys (*Saimiri oerstedi*) in Costa Rica. *Folia Primatologica, 49*(3-4), 151-167.

Bonadonna, G., Torti, V., Sorrentino, V., Randrianarison, R. M., Zaccagno, M., Gamba, M., . . . Giacoma, C. (2017). Territory exclusivity and intergroup encounters in the indris (Mammalia: Primates: Indridae: Indri indri) upon methodological tuning. *The European Zoological Journal, 84*(1), 238-251.

Boonratana, R. (1993). *The Ecology and Behaviour of the Proboscis Monkey (Nasalis larvatus) in the Lower Kinabatangan, Sabah*. Mahidol University, Bangkok.

Boonratana, R. (2000). Ranging behavior of proboscis monkeys (*Nasalis larvatus*) in the lower Kinabatangan, northern Borneo. *International Journal of Primatology, 21*, 497-518.

Boonratana, R., & Le, X. C. (1998). Preliminary observations of the ecology and behavior of the Tonkin snub-nosed monkey (*Rhinopithecus avunculus*) in Northern Vietnam. In N. G. Jablonski (Ed.), *The Natural History of the Doucs and Snub-Nosed Monkeys.* (pp. 207-217). Singapore: World Scientific Press.

Bravo, S. P., & Sallenave, A. (2003). Foraging behavior and activity patterns of *Alouatta caraya* in the northeastern Argentinean flooded forest. *International Journal of Primatology, 24*(4), 825-846.

Brockelman, W. Y. (1984). Maintenance and evolution of social structure in gibbons. *The lesser apes: Evolutionary and behavioural biology*.

Brown, M. (2013). Food and range defence in group-living primates. *Animal Behaviour, 85*(4), 807-816.

Bryant, J. V., Zeng, X., Hong, X., Chatterjee, H. J., & Turvey, S. T. (2017). Spatiotemporal requirements of the Hainan gibbon: Does home range constrain recovery of the world's rarest ape? *American Journal of Primatology, 79*(3), e22617.

Butynski, T. M. (1990). Comparative ecology of blue monkeys (*Cercopithecus mitis*) in high‐and low‐density subpopulations. *Ecological Monographs, 60*(1), 1-26.

Buzzard, P., & Eckardt, W. (2007). The social systems of the guenons. *Cambridge Studies in Biological and Evolutionary Anthropology, 51*, 51.

Byrne, R., Whiten, A., & Henzi, S. (1987). One-male groups and intergroup interactions of mountain baboons. *International Journal of Primatology*, 8(6), 615-633.

Caillaud, D., Levrero, F., Gatti, S., Menard, N., & Raymond, M. (2008). Influence of male morphology on male mating status and behavior during interunit encounters in western lowland gorillas. *American Journal of Physical Anthropology, 135*, 379-388.

Caillaud, D., Ndagijimana, F., Giarrusso, A. J., Vecellio, V., & Stoinski, T. S. (2014). Mountain gorilla ranging patterns: Influence of group size and group dynamics. *American Journal of Primatology, 76*(8), 730-746.

Campbell, C. J., Fuentes, A., MacKinnon, K. C., Bearder, S. K., & Stumpf, R. M. (Eds.). (2011). *Primates in Perspective, 2nd Edition*. Oxford University Press.

Campera, M., Serra, V., Balestri, M., Barresi, M., Ravaolahy, M., Randriatafika, F., & Donati, G. (2014). Effects of habitat quality and seasonality on ranging patterns of collared brown lemur (*Eulemur collaris*) in littoral forest fragments. *International Journal of Primatology, 35*(5), 957-975.

Chapman, C., & Pavelka, M. (2005). Group size in folivorous primates: ecological constraints and the possible influence of social factors *Primates, 46*, 1-9

Cheney, D. L. (1981). Intergroup encounters among free-ranging vervet monkeys. *Folia primatologica, 35*(2-3), 124-146.

Cheney, D. L. (1987). Interactions and relationships between groups In B. B. Smuts, D. L. Cheney, R. M. Seyfarth, R. W. Wrangham, & T. T. Struhsaker (Eds.), *Primate Societies* (pp. 267-281). Chicago: University of Chicago Press

Cheney, D. L., & Seyfarth, R. M. (1977). Behaviour of adult and immature male baboons during inter-group encounters. *Nature, 269*(5627), 404-406.

Cheyne, S. M., Thompson, C. J. H., Phillips, A. C., Hill, R. M. C., & Limin, S. H. (2008). Density and population estimate of gibbons (*Hylobates albibarbis*) in the Sabangau catchment, Central Kalimantan, Indonesia. *Primates, 49*(1), 50-56.

Chiarello, A. G. (1993). Home range of the brown howler monkey, *Alouatta fusca*, in a forest fragment of southeastern Brazil. *Folia Primatologica, 60*(3), 173-175.

Chiarello, A. G. (1995). Role of loud calls in brown howlers, *Alouatta fusca*. *American Journal of Primatology, 36*(3), 213-222.

Chivers, D. J. (1974). The siamang in Malaya: A field study of a primate in tropical rain forest. *Contributions to Primatology*(4), I-IX, 1-335.

Chivers, D. J., & Raemaekers, J. J. (1980). Long-term changes in behaviour. In *Malayan forest primates* (pp. 209-260): Springer.

Choudhury, A. (2008). Ecology and behaviour of the pig-tailed macaque *Macaca nemestrina leonina* in some forests of Assam in North-East India. *Journal of the Bombay Natural History Society, 105*(3), 279-291.

Cooksey, K. E., Sanz, C., Massamba, J. M., Ebombi, T. F., Tebard, P., Magema, E., . . . Morgan, D. (2020). Socioecological factors influencing intergroup encounters in western lowland gorillas (*Gorilla gorilla gorilla*). *International Journal of Primatology*.

Cooper, M. A., Aureli, F., & Singh, M. (2004). Between-group encounters among bonnet macaques (*Macaca radiata*). *Behavioral Ecology and Sociobiology, 56*(3), 217-227.

Cowlishaw, G. (1995). Behavioural patterns in baboon group encounters: the role of resource competition and male reproductive strategies. *Behaviour, 132*(1-2), 75-86.

Crofoot, M. C. (2008). *Intergroup competition in white-faced capuchin monkeys (Cebus capucinus): Automated radio-telemetry reveals how intergroup relationships shape space-use and foraging success.* (Ph.D. thesis). Harvard University, Cambridge, MA.

Curtin, R. (1975). *The socio-ecology of the common langur, Presbytis entellus in the Nepal Himalaya.* (PhD thesis ). University of California, Berkeley.

Curtin, S. (1980). Dusky and banded leaf monkeys. In D. J. Chivers (Ed.), *Malayan Forest Primates. Ten Years' Study in Tropical Rain Forest* (pp. 107-145). New York: Plenum Press.

Curtis, D. J., & Zaramody, A. (1998). Group size, home range use, and seasonal variation in the ecology of *Eulemur mongoz*. *International Journal of Primatology, 19*(5), 811-835.

Dasilva, G. L. (1989). *The ecology of the western black and white colobus (Colobus polykomos polykomos Zimmerman 1780) on a riverine island in southeastern Sierra Leone.* (PhD Dissertation ). University of Oxford.

Davies, A. G. (1984). *An Ecological Study of he Red Leaf Monkey (Presbytis rubicunda) in the Dipterocarp Forest of Northern Borneo.* (Ph.D. dissertation). University of Cambridge.

Dawson, G. A. (1979). The use of time and space by the Panamanian tamarin, *Saguinus oedipus*. *Folia Primatologica, 31*(4), 253-284.

Day, R. T., & Elwood, R. W. (1999). Sleeping site selection by the golden‐handed tamarin *Saguinus midas midas*: the role of predation risk, proximity to feeding sites, and territorial defence. *Ethology, 105*(12), 1035-1051.

DeCasien, A. R., Williams, S. A., & Higham, J. P. (2017). Primate brain size is predicted by diet but not sociality. *Nature Ecology & Evolution*, *1*, 0112.

De La Torre, S., Campos, F., & De Vries, T. (1995). Home range and birth seasonality of *Saguinus nigricollis graellsi* in Ecuadorian Amazonia. *American Journal of Primatology, 37*(1), 39-56.

Deag, J. M. (1973). Intergroup encounters in the wild Barbary macaque *Macaca sylvanus* L. *Comparative ecology and behaviour of primates*.

Defler, T. R. (1982). A comparison of intergroup behavior in *Cebus albifrons* and *C. apella*. *Primates, 23*(3), 385-392.

Defler, T. R. (1996). Aspects of the ranging pattern in a group of wild woolly monkeys (*Lagothrix lagothricha*). *American Journal of Primatology, 38*(4), 289-302.

DeVore, I., & Hall, K. R. L. (1965). Baboon ecology. *I. DeVore (Ed.), Primate Behavior. Field Studies of Monkeys and Apes, New York etc.(Holt, Rinehart and Winston) 1965, pp. 20-53.*

Di Bitetti, M. S. (2001). Home-range use by the tufted capuchin monkey (*Cebus apella nigritus*) in a subtropical rainforest of Argentina. *Journal of Zoology, 253*(1), 33-45.

Di Fiore, A. (2003). Ranging behavior and foraging ecology of lowland woolly monkeys (*Lagothrix lagotricha poeppigii*) in Yasuni National Park, Ecuador. *American Journal of Primatology, 59*(2), 47-66.

Dias, L. G., & Strier, K. B. (2003). Effects of group size on ranging patterns in *Brachyteles arachnoides hypoxanthus*. *International Journal of Primatology, 24*(2), 209-221.

DiFiore, A. F. (1997). *Ecology and behavior of lowland woolly monkeys (Lagothrix lagotricha poeppigii, Atelinae) in Eastern Ecuador*: University of California, Davis.

Dittus, W. P. J. (1987). Group fusion among wild toque macaques: an extreme case of inter-group resource competition. *Behaviour*, 247-291.

Doran‐Sheehy, D. M., Greer, D., Mongo, P., & Schwindt, D. (2004). Impact of ecological and social factors on ranging in western gorillas. *American Journal of Primatology, 64*(2), 207-222.

Dunbar, R. I. M., & Dunbar, E. P. (1975). *Social Dynamics of Gelada Baboons*. Basel, Switzerland: Karger.

Ehlers Smith, D. A. (2014). Preliminary evidence for the hired guns hypothesis and indirect mate defense in a wild group of red langurs (*Presbytis rubicunda*) in Sabangau tropical peat-swamp forest, Central Kalimantan, Indonesian Borneo. *Asian Primates Journal, 4*(2), 2-15.

Ellefson, J. O. (1974). A natural history of white-handed gibbons in the Malayan peninsula. *Gibbon and Siamang*(3), 1-136.

Ellis, K., & Di Fiore, A. (2019). Variation in space use and social cohesion within and between four groups of woolly monkeys (*Lagothrix lagotricha poeppigii*) in relation to fruit availability and mating opportunities at the Tiputini Biodiversity Station, Ecuador. In *Movement Ecology of Neotropical Forest Mammals* (pp. 141-171): Springer.

Enstam, K. L., Isbell, L. A., & De Maar, T. W. (2002). Male demography, female mating behavior, and infanticide in wild patas monkeys (*Erythrocebus patas*). *International Journal of Primatology, 23*(1), 85-104.

Erb, W. M. (2012). *Male-male competition and loud calls in one-male groups of simakobu (Simias concolor).* State University of New York at Stony Brook,

Fashing, P. J. (2001). Activity and ranging patterns of guerezas in the Kakamega Forest: intergroup variation and implications for intragroup feeding competition *International Journal of Primatology, 22*, 549-577.

Fashing, P. J. (2001). Male and female strategies during inter-group encounters in guerezas (*Colobus guereza*): evidence for resource defence mediated through males and a comparison with other primates. *Behavioral Ecology and Sociobiology, 50*, 219-230.

Ferrari, S. F., Kátia, H., Corrêa, M., & Coutinho, P. E. G. (1996). Ecology of the “southern” marmosets (*Callithrix aurita* and *Callithrix flaviceps*). In *Adaptive radiations of neotropical primates* (pp. 157-171): Springer.

Fischer, J., Kopp, G. H., Dal Pesco, F., Goffe, A., Hammerschmidt, K., Kalbitzer, U., . . . Zinner, D. (2017). Charting the neglected West: The social system of Guinea baboons. *American Journal of Physical Anthropology 162*, 15-31.

Fleury, M., & Gautier-Hion, A. (1999). Seminomadic ranging in a population of black colobus (*Colobus satanas*) in Gabon and its ecological correlates. *International Journal of Primatology, 20*, 491-509.

Forcina, G., Vallet, D., Le Gouar, P. J., Bernardo-Madrid, R., Illera, G., Molina-Vacas, G., . . . Bermejo, M. (2019). From groups to communities in western lowland gorillas. *Proceedings of the Royal Society B: Biological Sciences, 286*, 20182019.

Fuentes, A. (1996). Feeding and ranging in the Mentawai Island langur (*Presbytis potenziani*). *International Journal of Primatology, 17*, 525-548.

Gabow, S. L. (1973). Dominance order reversal between two groups of free-ranging rhesus monkeys. *Primates*.

Garber, P. A. (1988). Diet, foraging patterns, and resource defense in a mixed species troop of *Saguinus mystax* and *Saguinus fuscicollis* in Amazonian Peru. *Behaviour, 105*(1-2), 18-34.

Garber, P. A., Pruetz, J., & Isaacson, J. (1993). Patterns of range use, range defense, and intergroup spacing in moustached tamarin monkeys (*Saguinus mystax*). *Primates, 34*(1), 11-25.

Gatti, S., Levrero, F., Menard, N., & Gautier-Hion, A. (2004). Population and group structure of western lowland gorillas (*Gorilla gorilla gorilla*) at Lokoue, Republic of Congo. *American Journal of Primatology, 63*, 111-123.

Gautier‐Hion, A., & Gautier, J. P. (1978). Le singe de Brazza: une strategic originale. *Zeitschrift für Tierpsychologie, 46*(1), 84-104.

Gennuso, M. S., Brividoro, M., Pavé, R., Raño, M., & Kowalewski, M. (2018). Social play among black and gold howler monkey (Alouatta caraya) immatures during intergroup encounters. *American journal of primatology, 80*(9), e22909.

Gerber, B. D., Arrigo-Nelson, S., Karpanty, S. M., Kotschwar, M., & Wright, P. C. (2012). Spatial ecology of the endangered Milne-Edwards’ Sifaka (*Propithecus edwardsi*): Do logging and season affect home range and daily ranging patterns? *International Journal of Primatology, 33*(2), 305-321.

Gibson, L., & Koenig, A. (2012). Neighboring groups and habitat edges modulate range use in Phayre’s leaf monkeys (*Trachypithecus phayrei crepusculus*). *Behavioral Ecology and Sociobiology, 66*, 633-643.

Gittins, S. P. (1980). Territorial behavior in the agile gibbon. *International Journal of Primatology, 1*(WI 017433), 381-399.

Gómez-Posada, C., Martínez, J., Giraldo, P., & Kattan, G. H. (2007). Density, habitat use, and ranging patterns of red howler monkeys in a Colombian Andean forest. *Neotropical Primates, 14*(1), 2-10.

Goodall, J. (1979). Inter-community interactions in the chimpanzee population of the Gombe National Park. *The great apes*.

Gordon, A. D. (2006). Scaling of size and dimorphism in primates II: macroevolution. *International Journal of Primatology*, 27(1), 63-105.

Gould, L., & Overdorff, D. J. (2002). Adult male scent-marking in Lemur catta and Eulemur fulvus rufus. *International Journal of Primatology, 23*(3), 575-586.

Greenway, K. (2015). *Threat and display: reproductive competition in wild male Western Gorillas (Gorilla gorilla).* PhD thesis, School of Anthropology and Conservation. University of Kent.

Grueter, C. C. (2015). Home range overlap as a driver of intelligence in primates. *American Journal of Primatology, 77*, 418-424.

Grueter, C. C., Li, D., Ren, B., Wei, F., & Li, M. (2017). Deciphering the social organization and structure of wild Yunnan snub-nosed monkeys (*Rhinopithecus bieti*). *Folia Primatologica, 88*, 358-383.

Grueter, C. C. (2009). *Determinants of Modular Societies in Snub-nosed Monkeys (Rhinopithecus bieti) and other Colobines* [Ph.D. dissertation, University of Zurich]. Zurich, Switzerland.

Grueter, C. C., Erb, W., Ulibarri, L., & Matsuda, I. (2022). Ecology and behaviour of odd-nosed colobines. In I. Matsuda, C. C. Grueter, & J. A. Teichroeb (Eds.), *The Colobines: Natural History, Behaviour and Ecological Diversity* (pp. 156-185). Cambridge University Press.

Grueter, C. C., Isler, K., & Dixson, B. J. (2015). Are badges of status adaptive in large complex primate groups? *Evolution and Human Behavior*, *36*, 398-406.

Grueter, C. C., & van Schaik, C. P. (2009). Sexual size dimorphism in Asian colobines revisited. *American Journal of Primatology*, *71*, 609-616.

Grueter, C. C., & van Schaik, C. P. (2010). Evolutionary determinants of modular societies in colobines. *Behavioral Ecology*, *21*, 63-71.

Gurmaya, K. J. (1986). Ecology and behavior of *Presbytis thomasi* in Northern Sumatra. *Primates, 27*, 151-172.

Hamilton Iii, W. J., Buskirk, R. E., & Buskirk, W. H. (1975). Chacma baboon tactics during intertroop encounters. *Journal of Mammalogy, 56*(4), 857-870.

Hanya, G., Matsubara, M., Hayaishi, S., Zamma, K., Yoshihiro, S., Kanaoka, M. M., . . . Tsuriya, Y. (2008). Food conditions, competitive regime, and female social relationships in Japanese macaques: within-population variation on Yakushima. *Primates, 49*(2), 116-125.

Harding, R. S. O. (1976). Ranging patterns of a troop of baboons (*Papio anubis*) in Kenya. *Folia Primatologica, 25*(2-3), 143-185.

Harris, T. (2006). Between-group contest competition for food in a highly folivorous population of black and white colobus monkeys (*Colobus guereza*). *Behavioral Ecology and Sociobiology, 61*, 317-329.

Harrison, M. J. S. (1983). Territorial behavior in the green monkey, *Cercopithecus sabaeus*: seasonal defense of local food supplies. *Behavioral Ecology and Sociobiology, 12*, 85-94.

Hashimoto, C., Tashiro, Y., Kimura, D., Enomoto, T., Ingmanson, E. J., Idani, G. i., & Furuichi, T. (1998). Habitat use and ranging of wild bonobos (*Pan paniscus*) at Wamba. *International Journal of Primatology, 19*(6), 1045-1060.

Hausfater, G. (1972). Intergroup behavior of free-ranging rhesus monkeys (*Macaca mulatta*). *Folia Primatologica, 18*(1-2), 78-107.

Hendershott, R., Rawson, B., & Behie, A. (2018). Home range size and habitat use by Cat Ba Langurs (*Trachypithecus poliocephalus*) in a disturbed and fragmented habitat. *International Journal of Primatology, 39*(4), 547-566.

Herbinger, I., Boesch, C., & Rothe, H. (2001). Territory characteristics among three neighboring chimpanzee communities *International Journal of Primatology, 22*, 143-167.

Hoang, M. D. (2007). *Ecology and Conservation Status of the Black-Shanked Douc (Pygathrix nigripes) in Nui Cha and Phuoc Binh National Parks, Ninh Thuan Province, Vietnam.* (Ph.D. dissertation). University of Queensland,

Hoffman, T. S., & O'Riain, M. J. (2012). Troop Size and Human‐Modified Habitat Affect the Ranging Patterns of a Chacma Baboon Population in the C ape P eninsula, S outh A frica. *American Journal of Primatology, 74*(9), 853-863.

Hohmann, G., & Fruth, B. (2002). Dynamics in social organization of bonobos (*Pan paniscus*). In C. Boesch, G. Hohmann, & L. F. Marchant (Eds.), *Behavioural diversity in chimpanzees and bonobos* (pp. 138-150). New York: Cambridge University Press.

Holmes, S. M., Louis Jr, E. E., & Johnson, S. E. (2019). Range overlap and spatiotemporal relationships of frugivorous lemurs at Kianjavato, Madagascar. *Animal Behaviour, 155*, 53-65.

Hopkins, M. E. (2011). Mantled howler (*Alouatta palliata*) arboreal pathway networks: relative impacts of resource availability and forest structure. *International Journal of Primatology, 32*(1), 238-258.

Hopkins, M. E. (2013). Relative dominance and resource availability mediate mantled howler (*Alouatta palliata*) spatial responses to neighbors’ loud calls. *International Journal of Primatology, 34*(5), 1032-1054.

Hrdy, S. (1977). *The langurs of Abu - female and male strategies of reproduction*. Cambridge: Harvard University Press.

Idani, G. (1990). Relations between unit-groups of bonobos at Wamba, Zaire: encounters and temporary fusions. *African Study Monographs, 11*, 153-186.

Isbell, L. A., Bidner, L. R., Loftus, J. C., Kimuyu, D. M., & Young, T. P. (2021). Absentee owners and overlapping home ranges in a territorial species. *Behavioral Ecology and Sociobiology, 75*(1), 1-14.

Isbell, L. A., Cheney, D. L., & Seyfarth, R. M. (1990). Costs and benefits of home range shifts among vervet monkeys (*Cercopithecus aethiops*) in Amboseli National Park, Kenya. *Behavioral Ecology and Sociobiology, 27*, 351-358.

Isler, K., Kirk, E. C., Miller, J. M. A., Albrecht, G. A., Gelvin, B. R., & Martin, R. D. (2008). Endocranial volumes of primate species: scaling analyses using a comprehensive and reliable data set. *Journal of Human Evolution*, *55(6)*, 967-978.

Jay, P. (1965). The common langur of north India. In *Primate Behavior. Field Studies of Monkeys and Apes* (pp. 197-249). New York: Holt, Rinehart & Winston.

Johns, A. (1983). *Ecological Effects of Selective Logging in a West Malaysian Rain Forest*. Cambridge: Cambridge Universiy Press

Jolly, A. (1966). Lemur behavior.

Jolly, A., Rasamimanana, H. R., Kinnaird, M. F., O’Brien, T. G., Crowley, H. M., Harcourt, C. S., . . . Davidson, J. M. (1993). Territoriality in Lemur catta groups during the birth season at Berenty, Madagascar. In *Lemur social systems and their ecological basis* (pp. 85-109): Springer.

Kappeler, M. (1984). Vocal bouts and territorial maintenance in the moloch gibbon. *The lesser apes: Evolutionary and behavioral biology*.

Kavanagh, M. (1981). Variable territoriality among tantalus monkeys in Cameroon. *Folia Primatologica, 36*(1-2), 76-98.

Kawanaka, K. (1973). Intertroop relationships among Japanese monkeys. *Primates, 14*(2), 113-159.

Kinnaird, M. F. (1992). Variable resource defense by the Tana River crested mangabey *Behavioral Ecology and Sociobiology, 31*, 115-122

Kinzey, W. G., & Robinson, J. G. (1983). Intergroup loud calls, range size, and spacing in *Callicebus torquatus*. *American Journal of Physical Anthropology, 60*(4), 539-544.

Kitchen, D., Cheney, D., & Seyfarth, R. (2004). Factors mediating inter-group encounters in savannah baboons (*Papio cynocephalus ursinus*). *Behaviour*, 141(2), 197-218.

Koch, F. (2015). *Intergroup relationships in Verreaux’s sifakas (Propithecus verreauxi).* Georg-August-Universität Göttingen.

Kool, K. M. (1989). *Behavioural ecology of the silver leaf monkey, Trachypithecus auratus sondaicus, in the Pangandaran Nature Reserve, West Java, Indonesia.* (Ph.D. thesis). University of New South Wales, Sydney.

Korstjens, A., Nijssen, E., & Noë, R. (2005). Intergroup relationships in western black-and-white colobus, *Colobus polykomos polykomos*. *International Journal of Primatology, 26*, 1267-1289

Korstjens, A. H. (2001). *The mob, the secret sorority, and the phantoms.* (PhD Dissertation). Utrech University Utrecht.

Korstjens, A. H., & Noe, R. (2004). Mating system of an exceptional primate, the olive colobus (*Procolobus verus*). *American Journal of Primatology, 62(4)*, 261-273.

Kowalewski, M. M., & Garber, P. A. (2015). Solving the collective action problem during intergroup encounters: the case of black and gold howler monkeys (*Alouatta caraya*). In *Howler Monkeys* (pp. 165-189): Springer.

Kumara, H. N., Singh, M., Sharma, A. K., Santhosh, K., & Pal, A. (2014). Impact of forest fragment size on between-group encounters in lion-tailed macaques. *Primates, 55*(4), 543-548.

Kummer, H. (1968). *Social Organization of Hamadryas Baboons: A Field Study*. Chicago: The University of Chicago Press.

Lawes, M. J., & Henzi, S. P. (1995). Inter-group encounters in blue monkeys: how territorial must a territorial species be? *Animal Behaviour*.

Layton, R., O’Hara, S., & Bilsborough, A. (2012). Antiquity and social functions of multi-level social organisation among human hunter-gatherers. *International Journal of Primatology*, *33*, 1215-1245.

Lazaro-Perea, C. (2001). Intergroups interactions in wild common marmosets, *Callithrix jacchus*: territorial defence and assessment of neighbours. *Animal Behaviour, 62*, 11-21.

Levrero, F. (2005). *Structure d'une population de gorilles (Gorilla g. gorilla) visitant une clairière forestière: nature et rôle des rencontres intergroupes dans sa dynamique.* Rennes 1,

Lewis, R. J., Sandel, A. A., Hilty, S., & Barnett, S. E. (2020). The collective action problem but not numerical superiority explains success in intergroup encounters in Verreaux's Sifaka (*Propithecus verreauxi*): Implications for individual participation and free-riding. *International Journal of Primatology, 41*(2).

Li, P., Garber, P. A., Bi, Y., Jin, K., Qi, X., & Zhou, J. (2022). Diverse grouping and mating strategies in the Critically Endangered Hainan gibbon (*Nomascus hainanus*). *Primates*, *63*(3), 237-243.

Li, Z., & Rogers, M. (2005). Habitat quality and range use of white-headed langurs in Fusui, China *Folia Primatologica, 76*, 185-195

Lima, M., Mendes, S. L., & Strier, K. B. (2019). Habitat use in a population of the northern muriqui (*Brachyteles hypoxanthus*). *International Journal of Primatology, 40*(4), 470-495.

Lindburg, D. G. (1971). The rhesus monkey in north India: an ecological and behavioral study. In L. A. Rosenblum (Ed.), *Primate behavior: developments in field and laboratory research. Volume 2* (pp. 1-106). New York: Academic Press.

Lindburg, D. G. (1977). Feeding behaviour and diet of rhesus monkeys (Macaca mulatta) in a Siwalik forest in North India. *Primate ecology*.

Magliocca, F., & Gautier-Hion, A. (2004). Inter-group encounters in western lowland gorillas at a forest clearing. *Folia Primatologica, 75*(6), 379-382.

Majolo, B., McFarland, R., Young, C., & Qarro, M. (2013). The effect of climatic factors on the activity budgets of Barbary macaques (*Macaca sylvanus*). *International Journal of Primatology*.

Majolo, B., Ventura, R., & Koyama, N. F. (2005). Sex, rank and age differences in the Japanese macaque (*Macaca fuscata yakui*) participation in inter‐group encounters. *Ethology, 111*(5), 455-468.

Marsh, C. (1979). Comparative aspects of social organization in the Tana River red colobus, *Colobus badius rufomitratus*. *Zeitschrift für Tierpsychologie*, *51*, 337-362.

Martínez-Íñigo, L., Baas, P., Klein, H., Pika, S., & Deschner, T. (2021). Intercommunity interactions and killings in central chimpanzees (*Pan troglodytes troglodytes*) from Loango National Park, Gabon. *Primates*, 1-14.

Maruhashi, T., Saito, C., & Agetsuma, N. (1998). Home range structure and inter-group competition for land of Japanese macaques in evergreen and deciduous forests. *Primates, 39*(3), 291-301.

Mason, W. A. (1968). Use of space by Callicebus groups. *Primates: Studies in adaptation and variability*.

Matthews, L. J. (2009). Activity patterns, home range size, and intergroup encounters in Cebus albifrons support existing models of capuchin socioecology. *International Journal of Primatology, 30*(5), 709-728.

McKey, D., & Waterman, P. (1982). Ranging behavior of a group of black colobus (*Colobus satanas*) in the Douala-Edea Reserve, Cameroon *Folia Primatologica, 39*, 264-304.

McKey, D. B. (1979). *Plant Chemical Defenses And The Feeding And Ranging Behavior Of Colobus Monkeys In African Rainforests*: University of Michigan.

Megantara, E. (1989). Ecology, Behavior and Sociality of *Presbytis femoralis* in Eastcentral Sumatra. In A. Ehara & S. Kawamura (Eds.), *Comparative Primatology Monographs* (Vol. Vol. 2, pp. 171-301 ). Padjadjaran University of Padjadjaran, India

Mehlman, P. T., & Parkhill, R. S. (1988). Intergroup interactions in wild barbary macaques (*Macaca sylvanus*), Ghomaran Rif Mountains, Morocco. *American Journal of Primatology, 15*(1), 31-44.

Miller, A., Uddin, S., Judge, D. S., Kaplin, B., Ndayishimiye, D., Uwingeneye, G., & Grueter, C. C. (2020). Spatiotemporal association patterns in a supergroup of Rwenzori black‐and‐white colobus (*Colobus angolensis ruwenzorii*) are consistent with a multilevel society. *American Journal of Primatology, 82(6), e23127.*

Mirville, M. O., Ridley, A. R., Samedi, J. P. M., Vecellio, V., Ndagijimana, F., Stoinski, T. S., & Grueter, C. C. (2018). Low familiarity and similar ‘group strength’ between opponents increase the intensity of intergroup interactions in mountain gorillas (*Gorilla beringei beringei*). *Behavioral Ecology and Sociobiology, 72*, 178.

Mitani, J. C. (1985). Gibbon song duets and intergroup spacing. *Behaviour*, 59-96.

Mitchell, A. H. (1994). *Ecology of Hose's Langur, Presbytis hosei, in Mixed Logged and Unlogged Dipterocarp Forest of Northeast Borneo.* (Ph.D. thesis). Yale University, New Haven.

Mitchell, C. L., Boinski, S., & Van Schaik, C. P. (1991). Competitive regimes and female bonding in two species of squirrel monkeys (*Saimiri oerstedi* and *S. sciureus*). *Behavioral Ecology and Sociobiology, 28*(1), 55-60.

Mittermeier, R. A., & Wilson, D. E. (2013). Handbook of the mammals of the world: Vol. 3: Primates.

Moore, R. S., Nekaris, K. A. I., & Eschmann, C. (2010). Habitat use by western purple-faced langurs *Trachypithecus vetulus nestor* (Colobinae) in a fragmented suburban landscape. *Endangered Species Research, 12*(3), 227-234.

Moscovice, L. R., Hohmann, G., Trumble, B. C., Fruth, B., & Jaeggi, A. V. (2022). Dominance or Tolerance? Causes and consequences of a period of increased intercommunity encounters among bonobos (*Pan paniscus*) at LuiKotale. *International Journal of Primatology*, 1-26.

Mukherjee, R. P., & Saha, S. S. (1974). The golden langurs (*Presbytis geei* Khajuria, 1956) of Assam. *Primates, 15*, 327-340.

Nakagawa, N. (1999). Differential habitat utilization by patas monkeys (Erythrocebus patas) and tantalus monkeys (*Cercopithecus aethiops tantalus*) living sympatrically in northern Cameroon. *American Journal of Primatology, 49*(3), 243-264.

Nakamichi, M., & Koyama, N. (1997). Social relationships among ring-tailed lemurs (*Lemur catta*) in two free-ranging troops at Berenty Reserve, Madagascar. *International Journal of Primatology, 18*(1), 73-93.

Newton, P. (1992). Feeding and ranging patterns of forest hanuman langurs (*Presbytis entellus*). *International Journal of Primatology, 13*, 245-285.

Newton, P. N., & Dunbar, R. I. M. (1994). Colobine monkey society. In A. G. Davies & J. F. Oates (Eds.), *Colobine Monkeys: Their Ecology, Behavior and Evolution* (pp. 311-346). New York: Cambridge University Press.

Nie, S., Xiang, Z., & Li, M. (2009). Preliminary report on the diet and social structure of gray snub-nosed monkeys (*Rhinopithecus brelichi*) at Yangaoping,Guizhou,China. *Acta Theriologica Sinica, 29*, 326-331.

Nievergelt, C. M., Mutschler, T., & Feistner, A. T. C. (1998). Group encounters and territoriality in wild Alaotran gentle lemurs (*Hapalemur griseus alaotrensis*). *American Journal of Primatology, 46*(3), 251-258.

Nijman, V. (2022). Ecology of sympatric and allopatric Presbytis and Trachypithecus langurs in Sundaland. In I. Matsuda, C. C. Grueter & J. A. Teichroeb (Eds.), *The Colobines: Natural History, Behaviour and Ecological Diversity*. Cambridge University Press.

O'Brien, T. G., & Kinnaird, M. F. (1997). Behavior, diet, and movements of the Sulawesi crested black macaque (*Macaca nigra*). *International Journal of Primatology, 18*(3), 321-351.

Oates, J. (1977a). The guereza and its food. In T. H. Clutton-Brock (Ed.), *Primate Ecology: Studies of Feeding and Ranging Behaviour in Lemurs, Monkeys and Apes* (pp. 275-321). New York: Academic Press.

Oates, J. (1977b). The social life of a black-and-white colobus monkey, Colobus guereza. *Zeitschrift für Tierpsychologie*, *45*, 1-60.

Oates, J. F. (1994). The natural history of African colobines. In A. G. Davies & J. F. Oates (Eds.), *Colobine Monkeys: Their Ecology, Behaviour and Evolution* (pp. 75-128). Cambridge: Cambridge University Press.

Oi, T. (1990). Population organization of wild pig-tailed macaques (*Macaca nemestrina nemestrina*) in West Sumatra. *Primates, 31*(1), 15-31.

Okamoto, K., & Matsumura, S. (2002). Intergroup encounters in wild moor macaques (*Macaca maurus*). *Primates, 43*(2), 119-125.

Oliveira, A. C. M., & Ferrari, S. F. (2008). Habitat exploitation by free-ranging *Saguinus niger* in eastern Amazonia. *International Journal of Primatology, 29*(6), 1499.

Ostner, J., Kappeler, P., & Heistermann, M. (2008). Androgen and glucocorticoid levels reflect seasonally occurring social challenges in male redfronted lemurs (Eulemur fulvus rufus). *Behavioral Ecology and Sociobiology, 62*(4), 627-638.

Overdorff, D. J., & Tecot, S. R. (2006). Social pair-bonding and resource defense in wild red-bellied lemurs (*Eulemur rubriventer*). In *Lemurs* (pp. 235-254): Springer.

Pal, A., Kumara, H. N., Mishra, P. S., Velankar, A. D., & Singh, M. (2018). Between-group encounters in Nicobar long-tailed macaque (*Macaca fascicularis umbrosus*). *Ethology Ecology & Evolution, 30*(6), 582-599.

Palacios, E., & Rodriguez, A. (2001). Ranging pattern and use of space in a group of red howler monkeys (*Alouatta seniculus*) in a southeastern Colombian rainforest. *American Journal of Primatology: Official Journal of the American Society of Primatologists, 55*(4), 233-251.

Palma, A. C., Vélez, A., Gómez‐Posada, C., López, H., Zárate, D. A., & Stevenson, P. R. (2011). Use of space, activity patterns, and foraging behavior of red howler monkeys (Alouatta seniculus) in an Andean forest fragment in Colombia. *American Journal of Primatology, 73*(10), 1062-1071.

Palminteri, S., & Peres, C. A. (2012). Habitat selection and use of space by bald-faced sakis (*Pithecia irrorata*) in Southwestern Amazonia: Lessons from a multiyear, multigroup study. *International Journal of Primatology, 33*(2), 401-417.

Palminteri, S., Powell, G. V., & Peres, C. A. (2012). Advantages of granivory in seasonal environments: feeding ecology of an arboreal seed predator in Amazonian forests. *Oikos, 121*(11), 1896-1904.

Palminteri, S., Powell, G. V. N., & Peres, C. A. (2016). Determinants of spatial behavior of a tropical forest seed predator: the roles of optimal foraging, dietary diversification, and home range defense. *American Journal of Primatology, 78*(5), 523-533.

Parnell, R. J. (2002). The social structure and behaviour of western lowland gorillas (*Gorilla gorilla gorilla*) at Mbeli Bai, Republic of Congo.

Patzelt, A., Kopp, G. H., Ndao, I., Kalbitzer, U., Zinner, D., & Fischer, J. (2014). Male tolerance and male-male bonds in a multilevel primate society. *Proceedings of the National Academy of Sciences of the United States of America*, *111*, 14740-14745.

Payne, H. F. P., Lawes, M. J., & Henzi, S. P. (2003). Competition and the exchange of grooming among female samango monkeys (*Cercopithecus mitis erythrarchus*). *Behaviour, 140*, 453-471.

Peres, C. A. (1989). Costs and benefits of territorial defense in wild golden lion tamarins, *Leontopithecus rosalia*. *Behavioral Ecology and Sociobiology, 25*(3), 227-233.

Peres, C. A. (1992). Consequences of joint-territoriality in a mixed-species group of tamarin monkeys. *Behaviour, 123*(3-4), 220-246.

Peres, CA. (2000). Territorial defense and the ecology of group movements in small-bodied neotropical primates. In S. Boinski, & PA. Garber (Eds.), *On the Move: How and Why Animals Travel in Groups* (pp. 100-123). University of Chicago Press.

Perry, S. (1996). Intergroup encounters in wild white-faced capuchins (*Cebus capucinus*). *International Journal of Primatology, 17*, 309-330.

Pichon, C., & Simmen, B. (2015). Energy management in crowned sifakas (*Propithecus coronatus*) and the timing of reproduction in a seasonal environment. *American Journal of Physical Anthropology*, *158*(2), 269-278.

Pinto, A. C. B., Azevedo-Ramos, C., & de Carvalho Jr, O. (2003). Activity patterns and diet of the howler monkey *Alouatta belzebul* in areas of logged and unlogged forest in Eastern Amazonia. *Animal Biodiversity and Conservation, 26*(2), 39-49.

Plavcan, J. M., & Ruff, C. B. (2008). Canine size, shape, and bending strength in primates and carnivores. *American Journal of Physical Anthropology*, *136*(1), 65-84.

Poirier, F. E. (1968). Analysis of a Nilgiri langur (*Presbytis johnii*) home range change *Primates, 9*, 29-43

Poirier, F. E. (1970). The Nilgiri langur (*Presbytis johnii*) of South India. In L. Rosenblum (Ed.), *Primate Behavior: Developments in Field and Laboratory Research, Vol. 1* (pp. 251-383). New York: Academic Press.

Pollock, J. I. (1975). Field observations on Indri indri: a preliminary report. In *Lemur biology* (pp. 287-311): Springer.

Pollock, J. I. (1979). Spatial distribution and ranging behavior in lemurs. *The study of prosimian behavior*, 359-409.

Pombo, A. R., Waltert, M., Mansjoer, S. S., Mardiastuti, A., & Mühlenberg, M. (2004). Home range, diet and behaviour of the Tonkean macaque (*Macaca tonkeana*) in Lore Lindu National Park, Sulawesi. In *Land use, nature conservation and the stability of rainforest margins in Southeast Asia* (pp. 313-325): Springer.

Pontes, A. R. M., & da Cruz, M. A. O. M. (1995). Home range, intergroup transfers, and reproductive status of common marmosets *Callithrix jacchus* in a forest fragment in north-eastern Brazil. *Primates, 36*(3), 335-347.

Porter, L. M., & Garber, P. A. (2010). Mycophagy and its influence on habitat use and ranging patterns in Callimico goeldii. *American Journal of Physical Anthropology, 142*(3), 468-475.

Powzyk, J. A. (1997). *The socio-ecology of two sympatric Indrids. Propithecus diadema diadema and Indri indri: A comparison of feeding strategies and their possible repercussions on species-specific behaviors*: Duke University.

Price, E. C., & Piedade, H. M. (2001). Ranging behavior and intraspecific relationships of masked titi monkeys (*Callicebus personatus personatus*). *American Journal of Primatology: Official Journal of the American Society of Primatologists, 53*(2), 87-92.

Qi, X., Garber, P. A., Ji, W., Huang, Z., Huang, K., Zhang, P., . . . Li, B. (2014). Satellite telemetry and social modeling offer new insights into the origin of primate multilevel societies. *Nature Communications, 5*, 5296.

Quintana-Morales, P. C., Morales-Mávil, J. E., Escobar-Aliaga, M., & Bravo-Xicotencatl, R. (2017). Use of space in two neighboring groups of the howler monkey *Alouatta palliata mexicana* (Primates: Atelidae): overlap and home range size. *Therya, 8*(2), 91-97.

Raboy, B. E., & Dietz, J. M. (2004). Diet, foraging, and use of space in wild golden‐headed lion tamarins. *American Journal of Primatology, 63*(1), 1-15.

Ramanamisata, R., Pichon, C., Razafindraibe, H., & Simmen, B. (2014). Social behavior and dominance of the crowned sifaka (*Propithecus coronatus*) in northwestern Madagascar. *Primate Conservation, 2014*(28), 93-97.

Ramos-Fernández, G., & Ayala-Orozco, B. (2003). Population size and habitat use of spider monkeys at Punta Laguna, Mexico. In *Primates in fragments* (pp. 191-209): Springer.

Reichard, U., & Sommer, V. (1997). Group encounters in wild gibbons (*Hylobates lar*): agonism, affiliation, and the concept of infanticide. *Behaviour, 134*, 1135-1174.

Ren, R., Su, Y., Yan, K., Li, J., Yin, Z., Zhu, Z., . . . Hu, Y. (1998). Preliminary survey of the social organization of *Rhinopithecus roxellana* in Shennongjia National Natural Reserve, Hubei, China. In N. G. Jablonski (Ed.), *The Natural History of the Doucs and Snub-Nosed Monkeys* (pp. 269-279). Singapore: World Scientific Press.

Richard, A. (1977). The feeding behaviour of *Propithecus verreauxi*. In *Primate ecology: Studies of feeding and ranging behavior in lemurs, monkey and apes* (pp. 71-96): Elsevier.

Rigamonti, M. M. (1993). Home range and diet in red ruffed lemurs (*Varecia variegata rubra*) on the Masoala Peninsula, Madagascar. In *Lemur social systems and their ecological basis* (pp. 25-39): Springer.

Ripley, S. (1967). Intertroop encounters among Ceylon gray langurs (*Presbytis entellus*). *Social communication among primates*.

Robbins, M. M., & Sawyer, S. C. (2007). Intergroup encounters in mountain gorillas of Bwindi Impenetrable National Park, Uganda. *Behaviour, 144*, 1497-1519.

Robinson, J. G. (1979). Vocal regulation of use of space by groups of titi monkeys *Callicebus moloch*. *Behavioral Ecology and Sociobiology, 5*(1), 1-15.

Robinson, J. G. (1981). Vocal regulation of inter-and intragroup spacing during boundary encounters in the titi monkey, *Callicebus moloch*. *Primates, 22*(2), 161-172.

Robinson, J. G. (1986). Seasonal variation in use of time and space by the wedge-capped capuchin monkey, Cebus olivaceus: implications for foraging theory. *Smithsonian Contributions to Zoology*.

Robinson, J. G. (1988). Group size in wedge-capped capuchin monkeys *Cebus olivaceus* and the reproductive success of males and females. *Behavioral Ecology and Sociobiology, 23*(3), 187-197.

Roth, A. M., & Cords, M. (2016). Effects of group size and contest location on the outcome and intensity of intergroup contests in wild blue monkeys. *Animal Behaviour, 113*, 49-58.

Rowe, N., & Myers, M. (2016). *All the world's primates* (Vol. 777). Pogonias Press Charlestown.

Ruhiyat, Y. (1983). Socio-ecological study of *Presbytis aygula* in West Java. *Primates, 24*, 344-359.

Rylands, A. B. (1986). Ranging behaviour and habitat preference of a wild marmoset group, *Callithrix humeralifer* (Callitrichidae, Primates). *Journal of Zoology, 210*(4), 489-514.

Rylands, A. B. (1989). Sympatric Brazilian callitrichids: the black tufted-ear marmoset, *Callithrix kuhli*, and the golden-headed lion tamarin, *Leontopithecus chrysomelas*. *Journal of Human evolution, 18*(7), 679-695.

Saj, T. L., & Sicotte, P. (2007). Scramble competition among *Colobus vellerosus* at Boabeng-Fiema, Ghana. *International Journal of Primatology, 28*, 337-355.

Sakamaki, T., Ryu, H., Toda, K., Tokuyama, N., & Furuichi, T. (2018). Increased frequency of intergroup encounters in wild bonobos (*Pan paniscus*) around the yearly peak in fruit abundance at Wamba. *International Journal of Primatology, 39*, 685-704.

Salafsky, N. (1988). *The Foraging Patterns and Socioecology of the Kelasi (Presbytis rubicunda).* (MSc thesis). Harvard College, Cambridge, MA.

Samuni, L., Wegdell, F., & Surbeck, M. (2020). Behavioural diversity of bonobo prey preference as a potential cultural trait. *Elife, 9*, e59191.

Sangchantr, S. (2004). *Social organization and ecology of Mentawai leaf monkeys (Presbytis potenziani).* Columbia University.

Santhosh, K., Kumara, H. N., Velankar, A. D., & Sinha, A. (2015). Ranging behavior and resource use by lion-tailed macaques (*Macaca silenus*) in selectively logged forests. *International Journal of Primatology, 36*(2), 288-310.

Sato, H. (2013). Habitat shifting by the common brown lemur (Eulemur fulvus fulvus): a response to food scarcity. *Primates, 54*(3), 229-235.

Schneider, I., Tielen, I. H. M., Rode, J., Levelink, P., & Schrudde, D. (2010). Behavioral observations and notes on the vertical ranging pattern of the critically endangered Cat Ba langur (*Trachypithecus poliocephalus poliocephalus*) in Vietnam. *Primate Conservation*, *2010*(25), 111-117.

Schoof, V. A. M., & Jack, K. M. (2013). The association of intergroup encounters, dominance status, and fecal androgen and glucocorticoid profiles in wild male white‐faced capuchins (*Cebus capucinus*). *American Journal of Primatology, 75*(2), 107-115.

Seiler, N., Boesch, C., Mundry, R., Stephens, C., & Robbins, M. M. (2017). Space partitioning in wild, non-territorial mountain gorillas: the impact of food and neighbours. *Royal Society Open Science, 4*(11), 170720.

Sekulic, R. (1982a). Daily and seasonal patterns of roaring and spacing in four red howler *Alouatta seniculus* troops. *Folia Primatologica, 39*(1-2), 22-48.

Sekulic, R. (1982b). The function of howling in red howler monkeys (*Alouatta seniculus*). *Behaviour*, 38-54.

Senf, M. J. (2009). *Interspecific and integroup interactions of mantled howling monkeys (Alouatta palliata) in primary versus secondary forest at El Zota Biological Field Station, Costa Rica*: Iowa State University.

Shaffer, C. A. (2012). *Ranging behavior, group cohesiveness, and patch use in northern bearded sakis (Chiropotes sagulatus) in Guyana*: Washington University in St. Louis.

Shanee, S. (2014). Ranging behaviour, daily path lengths, diet and habitat use of yellow-tailed woolly monkeys (*Lagothrix flavicauda*) at La Esperanza, Peru. In *The woolly monkey* (pp. 167-185): Springer.

Shimooka, Y. (2005). Sexual differences in ranging of *Ateles belzebuth belzebuth* at La Macarena, Colombia. *International Journal of Primatology, 26*(2), 385-406.

Shopland, J. M. (1982). An intergroup encounter with fatal consequences in yellow baboons (*Papio cynocephalus*). *American Journal of Primatology, 3*(1‐4), 263-266.

Sicotte, P. (1993). Inter-group encounters and female transfer in mountain gorillas: Influence of group composition on male behavior. *American Journal of Primatology, 30(1)*, 21-36.

Sicotte, P., & Macintosh, A. (2004). Inter-group encounters and male incursions in *Colobus vellerosus* in central Ghana. *Behaviour*(141(5)), 533-553.

Siex, K. S. (2003). *Effects of population compression on the demography, ecology, and behavior of the Zanzibar red colobus monkey (Procolobus kirkii).* (PhD). Duke University,

Slater, K., Barrett, A., & Brown, L. R. (2018). Home range utilization by chacma baboon (*Papio ursinus*) troops on Suikerbosrand Nature Reserve, South Africa. *PLoS ONE, 13*(3), e0194717.

Smith, R. J., & Jungers, W. L. (1997). Body mass in comparative primatology. *Journal of Human Evolution*, *32*, 523-559.

Soini, P. (1982). Ecology and population dynamics of the pygmy marmoset, Cebuella pygmaea. *Folia primatologica, 39*(1-2), 1-21.

Stanford, C. (1991). Social dynamics of of intergroup encounters in the capped langur (*Presbytis pileata*). *American Journal of Primatology, 25*, 35-47.

Stanford, C. B. (1991). The capped langur in Bangladesh: Behavioral ecology and reproductive tactics. *Contributions to Primatology, 26*, 1-179.

Starin, E. (1978). A preliminary investigation of home range use in the Gir Forest Langur *Primates, 19*, 551-568

Starin, E. D. (1991). *Socioecology of the Red Colobus Monkey in the Gambia with Particular Reference o Female-Male Differences and Transfer Patterns.* (Ph.D. thesis). City University of New York, New York.

Stead, S. M., & Teichroeb, J. A. (2019). A multi-level society comprised of one-male and multi-male core units in an African colobine (*Colobus angolensis ruwenzorii*). *PLoS ONE, 10*, e0217666.

Steenbeek, R. (1999a). *Female choice and male coercion in wild Thomas's langurs.* (Ph.D. thesis). University of Utrecht, Utrecht.

Steenbeek, R. (1999b). Tenure related changes in wild Thomas's langurs: I: Between-group interactions. *Behaviour, 136*, 595-625

Stojan-Dolar, M., & Heymann, E. W. (2010). Vigilance in a cooperatively breeding primate. *International Journal of Primatology, 31*(1), 95-116.

Stoltz, L. P., & Saayman, G. S. (1970). Ecology and behaviour of baboons in the northern Transvaal. *Annals of the Transvaal Museum, 26*(5), 99-143.

Strier, K. B. (1986). The behavior and ecology of the woolly spider monkey, or muriqui (*Brachyteles arachnoides* E. Geoffroy 1806).

Strier, K. B. (1987). Ranging behavior of woolly spider monkeys, or muriquis, *Brachyteles arachnoides*. *International Journal of Primatology, 8(6)*, 575-591.

Struhsaker, T. T. (1967a). Behavior of vervet monkeys (*Cercopithecus aethiops*). *University of California Publications of Zoology, 82*, 1-64.

Struhsaker, T. T. (1967b). Social structure among vervet monkeys (*Cercopithecus aethiops*). *Behaviour, 29*, 83-121.

Struhsaker, T. T. (1975). *The Red Colobus Monkey*. Chicago: The University of Chicago Press.

Struhsaker, T. T. (2010). *The Red Colobus Monkeys: Variation in Demography, Behavior, and Ecology of Endangered Species*. Oxford: Oxford University Press.

Struhsaker, T. T., & Leland, L. (1979). Socioecology of five sympatric monkey species in the Kibale Forest, Uganda. *Advances in the Study of Behavior*(9), 159-228.

Sugiura, H., Saito, C., Sato, S., Agetsuma, N., Takahashi, H., Tanaka, T., . . . Takahata, Y. (2000). Variation in intergroup encounters in two populations of Japanese macaques. *International Journal of Primatology, 21*(3), 519-535.

Sugiyama, Y. (1967). Social organization of Hanuman langurs. *Social communication among primates*, 221-236.

Sugiyama, Y. (1976). Characteristics of the ecology of he Himalayan langurs. *Journal of Human Evolution, 5*, 249-277.

Supriatna, J., Manullang, B., & Soekara, E. (1986). Group composition, home range, and diet of the maroon leaf monkey (*Presbytis rubicunda*) at Tanjung Puting Reserve, Central Kalimantan, Indonesia. *Primates, 27*, 185-190.

Suwanvecho, U., & Brockelman, W. Y. (2012). Interspecific territoriality in gibbons (*Hylobates lar* and *H. pileatus*) and its effects on the dynamics of interspecies contact zones. *Primates, 53*(1), 97-108.

Symington, M. M. (1988). Demography, ranging patterns, and activity budgets of black spider monkeys (*Ateles paniscus chamek*) in the Manu National Park, Peru. *American Journal of Primatology, 15*(1), 45-67.

Takasaki, H. (1981). Troop size, habitat quality, and home range area in Japanese macaques. *Behavioral Ecology and Sociobiology*(9), 277-281. Retrieved from PRINT (Diss - ranging)

Tan, C. L., Guo, S., & Li, B. (2007). Population structure and ranging patterns of *Rhinopithecus roxellana* in Zhouzhi National Reserve, Shaanxi, China. *International Journal of Primatology, 28*, 577-591.

Teichroeb, J. A., & Sicotte, P. (2018). Cascading competition: the seasonal strength of scramble influences between-group contest in a folivorous primate. *Behavioral Ecology and Sociobiology, 72*(1), 1-16.

Teichroeb, J. A., Adams, F. V., Khwaja, A., Stapelfeldt, K., & Stead, S. M. (2022). Tight quarters: ranging and feeding competition in a Colobus angolensis ruwenzorii multilevel society occupying a fragmented habitat. *Behavioral Ecology and Sociobiology*, *76*(5), 60.

Tenaza, R. R. (1975). Territory and monogamy among Kloss’ gibbons (Hylobates klossii) in Siberut Island, Indonesia. *Folia Primatologica, 24*(1), 60-80.

Tenaza, R. R., & Fuentes, A. (1995). Monandrous social organization of pigtailed langurs (*Simias concolor*) in the Pagai Islands, Indonesia. *Int J Primatol, 16*, 295-311

Terborgh, J. (1983). *Five New World Primates - A Study in Comparative Ecology* Princeton: Princeton University Press

Thompson, C. L., Norconk, M. A., & Whitten, P. L. (2012). Why fight? Selective forces favoring between-group aggression in a variably pair-living primate, the white-faced saki (Pithecia pithecia). *Behaviour, 149*(8), 795-820.

Ulibarri, L. R. (2013). *The Socioecology of Red-shanked Doucs (Pygathrix nemaeus) in Son Tra Nature Reserve, Vietnam.* (PhD thesis). University of Colorado, Boulder.

Van Belle, S., & Estrada, A. (2020). The influence of loud calls on intergroup spacing mechanism in black howler monkeys (*Alouatta pigra*). *International Journal of Primatology, 41*(2), 265-286.

Van Belle, S., Porter, A., Fernandez‐Duque, E., & Di Fiore, A. (2018). Ranging behavior and potential for territoriality in equatorial sakis (*Pithecia aequatorialis*) in Amazonian Ecuador. *American journal of physical anthropology, 167*(4), 701-712.

Van Belle, S., & Scarry, C. J. (2015). Individual participation in intergroup contests is mediated by numerical assessment strategies in black howler and tufted capuchin monkeys. *Philosophical Transactions of the Royal Society B: Biological Sciences, 370*(1683), 20150007.

Van Belle, S., Porter, A. M., Fernandez‐Duque, E., & Di Fiore, A. (2021). Ranging behavior and the potential for territoriality in pair‐living titi monkeys (*Plecturocebus discolor*). *American Journal of Primatology*, 83(5), e23225.

van Roosmalen, M. G. M. (1985). Habitat preferences, diet, feeding strategy and social organization of the black spider monkey [*Ateles paniscus paniscus* Linnaeus 1758] in Surinam. *Acta Amazonica, 15*, 7-238.

van Schaik, C. P., Assink, P. R., & Salafsky, N. (1992). Territorial behavior in southeast Asian langurs: resource defense or mate defense? *American Journal of Primatology, 26*, 233-242.

Vessey, S. H. (1968). Interactions between free-ranging groups of rhesus monkeys. *Folia Primatologica, 8*(3-4), 228-239.

Vogel, C. (1975). *Intergroup relations of Presbytis entellus in Kumaon Hills and Rajasthan (North India).* Paper presented at the 5th International Congress of Primatology.

Vogt, M. (2003). *Freilanduntersuchungen zur Ökologie und zum Verhalten von Trachypithecus auratus kohlbruggei (Haubenlanguren) im West-Bali-Nationalpark, Indonesien.* Eberhard-Karls-Universität, Tübingen.

Volampeno, M. S. N., Masters, J. C., & Downs, C. T. (2011). Home range size in the blue-eyed black lemur (*Eulemur flavifrons*): A comparison between dry and wet seasons. *Mammalian Biology, 76*(2), 157-164.

von Hippel, F. A. (1996). Interactions between overlapping multimale groups of black and white colobus monkeys (*Colobus guereza*) in the Kakamega Forest, Kenya. *American Journal of Primatology*(38), 193-209.

Wahome, J. M., Rowell, T. E., & Tsingalia, H. M. (1993). The natural history of de Brazza's monkey in Kenya. *International Journal of Primatology, 14*(3), 445-466.

Waser, P. M. (1976). Cercocebus albigena: Site attachment, avoidance and intergroup spacing. *American Naturalist, 110*, 911-935.

Watanabe, K. (1981). Variation in group composition and population density of the two sympatric Mentawaian leaf monkeys. *Primates, 22*, 145-160

Watts, D. P., & Mitani, J. C. (2001). Boundary patrols and intergroup encounters in wild chimpanzees. *Behaviour, 138*, 299-327.

Werre, J. (2000). *Ecology and Behavior of the Niger Delta Red Colobus (Procolobus badius epieni).* (PhD). City University, New York.

Wich, S., & Sterck, E. (2007). Familiarity and threat of opponenst determine variation in Thomas langur (*Presbytis thomasi*) male behaviour during between-group encounters. *Behaviour, 144*, 1583-1598.

Willems, E. P., Hellriegel, B., & van Schaik, C. P. (2013). The collective action problem in primate territory economics. *Proceedings of the Royal Society B, 280*, 20130081.

Williams-Guillén, K. (2003). *The behavioral ecology of mantled howling monkeys (Alouatta palliata) living in a Nicaraguan shade coffee plantation*: New York University.

Williams, J. M., Oehlert, G. W., Carlis, J. V., & Pusey, A. E. (2004). Why do male chimpanzees defend a group range? *Animal Behaviour, 68*(3), 523-532.

Xiang, Z., Xiao, W., Huo, S., & Li, M. (2013). Ranging pattern and population composition of *Rhinopithecus bieti* at Xiaochangdu: Implications for conservation. *Chinese Science Bulletin, 58*, 2212.

Yeager, C. P. (1989). *Proboscis Monkey (Nasalis larvatus) Social Organization and Ecology.* Ph.D. thesis. University of California Davis.

Yeager, C. P. (1990). Proboscis monkey (*Nasalis larvatus*) social organization: group structure. *American Journal of Primatology, 20*, 95-106.

Yeager, C. P. (1991). Proboscis monkey (*Nasalis larvatus*) social organization: Intergroup patterns of association. *American Journal of Primatology, 23*, 73-86.

Yi, Y., Fichtel, C., Ham, S., Jang, H., & Choe, J. C. (2020). Fighting for what it’s worth: participation and outcome of inter-group encounters in a pair-living primate, the Javan gibbon (*Hylobates moloch*). *Behavioral Ecology and Sociobiology, 74*(8), 1-15.

Yoshiba, K. (1968). Local and intergroup variability in ecology and social behavior of common Indian langurs. *Primates: Studies in adaptation and variability*.

Zhang, P., & Watanabe, K. (2012). Variation in intergroup encounters among two provisioned free-ranging populations of Japanese macaques Macaca fuscata. *Current Zoology, 58*(4), 517-524.

Zhang, P., Watanabe, K., Li, B., & Tan, C. L. (2006). Social organization of Sichuan snub-nosed monkeys (*Rhinopithecus roxellana*) in the Qinling Mounains, Central China *Primates, 47*, 374-382.

**Table S2.** Examples of how the scoring system was applied to quantify degrees of dimorphism in primate ornaments.

| **0** | **1** | **2** | **3** | **4** | **5** |
| --- | --- | --- | --- | --- | --- |
| **Hairy traits (e.g. capes, tufts, and beards)** | | | | | |
| *Saguinus imperator*: the  moustache is equally developed in both sexes | *Semnopithecus*  *schistaceus*: male facial hair slightly longer than in the female | *Colobus angolensis*: Tail not tufted in females | *Macaca fascicularis*: male has more  prominent cheek hair (‘whiskers’)  than female | *Pongo*  *pygmaeus*: male's beard is much larger than female's | *Homo sapiens*: male beard is markedly  more prominent than in females |
| **Fleshy traits** | | | | | |
| *Rhinopithecus bieti*: the bright pink lips are equally developed in both sexes |  |  | *Papio hamadryas*: male has red sex skin on rump, which resembles that of the female but differs in morphology from the female's swelling | *Pongo pygmaeus*: male has a fatty/fibrous  hump on top of head that is very sexually  dimorphic | *Pongo*  *pygmaeus*: male has  markedly larger cheek  flanges |
| **Colourful traits** | | | | | |
| *Macaca mulatta*: the red sexual skin on the rump and genitalia is very similar between the sexes | *Rhinopithecus*  *brelichi*: red hair on back of their head | *Cacajao calvus*: both sexes lose hair and have reddened facial skin that is accentuated in the male due to their larger temporal muscles | *Cercopithecus diana*: Blue scrotum | *Rhinopithecus*  *bieti*: pelage is much more contrastingly  coloured in males |  |

**Table S3.** The ornamentation scores and descriptions of the individual traits for each primate species used in the study.

| Species | Ornamentation score | Reference | Description |
| --- | --- | --- | --- |
| *Alouatta belzebul* | 2 | This study | Dusty red scrotum (2) |
| *Alouatta caraya* | 3 | Grueter et al. 2015 | Rust-red scrotum (2). Male’s beard larger than female’s (1) |
| *Alouatta guariba* | 1 | Grueter et al. 2015 | Male seems to have a slight beard (1) |
| *Alouatta palliata* | 2 | This study | Male has white pendulous scrotum (2) |
| *Alouatta pigra* | 0 | Grueter et al. 2015 |  |
| *Alouatta seniculus* | 4 | Grueter et al. 2015 | Long blackish beard (4) |
| *Ateles belzebuth* | 0 | Grueter et al. 2015 |  |
| *Ateles chamek* | 0 | This study |  |
| *Ateles geoffroyi* | 0 | Grueter et al. 2015 |  |
| *Ateles paniscus* | 0 | Dixson et al. 2005 |  |
| *Brachyteles hypoxanthus* | 1 | This study | Males have large conspicuous scrotum with dyspigmentation (1) |
| *Callicebus discolor* | 0 | This study |  |
| *Callicebus ornatus* | 0 | This study |  |
| *Callicebus personatus* | 0 | This study |  |
| *Callicebus lucifer* | 0 | This study |  |
| *Callimico goeldii* | 0 | Grueter et al. 2015 |  |
| *Callithrix jacchus* | 0 | Grueter et al. 2015 |  |
| *Callithrix aurita* | 0 | This study |  |
| *Cebuella pygmaea* | 0 | Grueter et al. 2015 |  |
| *Cebus albifrons* | 0 | Grueter et al. 2015 |  |
| *Cebus capucinus imitator* | 0 | Grueter et al. 2015 |  |
| *Cebus olivaceus* | 0 | Grueter et al. 2015 |  |
| *Cercocebus galeritus* | 0 | Grueter et al. 2015 |  |
| *Cercopithecus ascanius* | 0 | Grueter et al. 2015 |  |
| *Cercopithecus campbelli* | 0 | Grueter et al. 2015 |  |
| *Cercopithecus cephus* | 0 | Grueter et al. 2015 |  |
| *Cercopithecus diana* | 3 | Grueter et al. 2015 | Male has a blue scrotum (3) |
| *Cercopithecus mitis* | 2 | Grueter et al. 2015 | Male genitals slightly bluish (2) |
| *Cercopithecus neglectus* | 3 | Grueter et al. 2015 | Male has a blue scrotum (3) |
| *Cercopithecus nictitans* | 0 | Grueter et al. 2015 |  |
| *Cercopithecus petaurista* | 3 | This study | Male has blue scrotum (3) |
| *Chiropotes sagulatus* | 2 | This study | Male has a pinkish scrotum (2) |
| *Chlorocebus pygerythrus* | 4 | Grueter et al. 2015 | Male has red sex skin on penile shaft and blue on the scrotum (4) |
| *Chlorocebus sabaeus* | 3 | This study | Scrotum ranges from pale blue to bright blue (3) |
| *Chlorocebus tantalus* | 4 | This study | Sky blue scrotum surrounded by a tuft of orange hair (4) |
| *Colobus angolensis* | 2 | Grueter et al. 2015 | Tail not tufted in females (2) |
| *Colobus guereza* | 0 | Grueter et al. 2015 |  |
| *Colobus polykomos* | 0 | Grueter et al. 2015 |  |
| *Colobus satanas* | 0 | Grueter et al. 2015 |  |
| *Colobus vellerosus* | 0 | Grueter et al. 2015 |  |
| *Erythrocebus patas* | 4 | Grueter et al. 2015 | Blue scrotum and red anogenital field in male (lacking in female) (4) |
| *Eulemur collaris* | 2.5 | This study | Cheek ruffs of females are far less developed than those of males (2.5) |
| *Eulemur fulvus* | 0 | This study |  |
| *Eulemur flavifrons* | 0 | This study |  |
| *Eulemur macaco* | 0 | Lüpold et al. 2019 |  |
| *Eulemur mongoz* | 0 | This study |  |
| *Eulemur rubriventer* | 0 | Lüpold et al. 2019 |  |
| *Eulemur rufus* | 0 | This study |  |
| *Gorilla beringei* | 10 | Grueter et al. 2015 | Mature male has white saddle of hair on back: females lack this (5). Male has a pad of fibrous/fatty tissue on top of the head; this is lacking in female (5) |
| *Gorilla gorilla gorilla* | 10 | Grueter et al. 2015 | Mature male has white saddle of hair on back: females lack this (5). Male has a pad of fibrous/fatty tissue on top of the head; this is lacking in female (5) |
| *Hapalemur griseus alaotrensis* | 0 | Lüpold et al. 2019 |  |
| *Homo sapiens* | 10 | Grueter et al. 2015 | Beard is highly sexually dimorphic (5). Male pattern baldness (some men only) (2.5). Body hair generally greater in men than women (2.5) |
| *Hoolock hoolock* | 0 | This study |  |
| *Hylobates agilis* | 3 | Grueter et al. 2015 | Male has longish white cheek hair (3) |
| *Hylobates albibaris* | 2 | This study | Males often have a light genital tuft (2) |
| *Hylobates klossi* | 0 | Grueter et al. 2015 |  |
| *Hylobates lar* | 0 | Grueter et al. 2015 |  |
| *Hylobates moloch* | 0 | Grueter et al. 2015 |  |
| *Hylobates muelleri* | 2 | Grueter et al. 2015 | Male has incomplete face ring of hair (2) |
| *Hylobates pileatus* | 2.5 | Grueter et al. 2015 | White pubic tuft in males (2.5) |
| *Indri indri* | 0 | This study |  |
| *Lagothrix lagothricha* | 0 | Grueter et al. 2015 |  |
| *Lagothrix flavicauda* | 2 | Grueter et al. 2015 | Male has small yellow scrotal tuft (2) |
| *Lemur catta* | 0 | Lüpold et al. 2019 |  |
| *Leontopithecus chrysomelas* | 0 | This study |  |
| *Leontopithecus rosalia* | 0 | Grueter et al. 2015 |  |
| *Lophocebus albigena* | 0 | Grueter et al. 2015 |  |
| *Macaca fascicularis* | 3 | Grueter et al. 2015 | Male has more prominent cheek hair (‘whiskers’) than female (3) |
| *Macaca fuscata* | 0 | Grueter et al. 2015 |  |
| *Macaca leonina* | 2 | This study | Cheek buff ('lion mane') more pronounced in males (1). Pelage of adult females is somewhat shorter, paler, and drabber than that of males (1) |
| *Macaca maura* | 0 | This study |  |
| *Macaca mulatta* | 0 | This study |  |
| *Macaca nemestrina* | 0 | Grueter et al. 2015 |  |
| *Macaca nigra* | 0 | Grueter et al. 2015 |  |
| *Macaca radiata* | 0 | Grueter et al. 2015 |  |
| *Macaca silenus* | 0 | Grueter et al. 2015 |  |
| *Macaca sylvanus* | 0 | Grueter et al. 2015 |  |
| *Macaca tonkeana* | 0 | This study |  |
| *Mico intermedius* | 0 | This study |  |
| *Nasalis larvatus* | 9 | Grueter et al. 2015 | Adult male has a large, pendulous nose; female has a small nose (5). Male genitalia red (penis) and black (scrotum) in contrast to female (4) |
| *Nomascus hainanus* | 0 | This study |  |
| *Pan paniscus* | 0 | Grueter et al. 2015 |  |
| *Pan troglodytes* | 0 | Grueter et al. 2015 |  |
| *Papio anubis* | 4 | Grueter et al. 2015 | Mane more prominent in males (4) |
| *Papio cynocephalus* | 2 | Grueter et al. 2015 | Poorly developed mane in males (2) |
| *Papio hamadryas* | 13 | Grueter et al. 2015 | Male has long silver shoulder ‘cape’ of hair; female lacks this (5). Male has long silvery hair on sides of head; female lacks this (5). Male has red sex skin on rump, which is said to resemble that of the female, but which differs in its morphology from the female’s swelling (3) |
| *Papio papio* | 6 | Grueter et al. 2015 | Male: shoulder hair longer than in female (3). Male has longer hair on crown and sides of head than female (3) |
| *Papio ursinus* | 2 | Grueter et al. 2015 | Poorly developed mane in males (2) |
| *Piliocolobus badius* | 0 | Grueter et al. 2015 |  |
| *Piliocolobus epieni* | 0 | This study |  |
| *Piliocolobus rufomitratus* | 0 | This study |  |
| *Piliocolobus temminckii* | 0 | This study |  |
| *Piliocolobus tephrosceles* | 0 | This study |  |
| *Piliocolobus kirkii* | 0 | This study |  |
| *Pithecia aequatorialis* | 0 | This study |  |
| *Pithecia irrorata* | 2 | This study | Males have dark pink hairless facial skin (2) |
| *Pithecia pithecia* | 0 | Grueter et al. 2015 |  |
| *Presbytis comata* | 0 | Grueter et al. 2015 |  |
| *Presbytis femoralis* | 0 | This study |  |
| *Presbytis potenziani* | 1 | This study | Male has a white scrotum (1) |
| *Presbytis rubicunda* | 0 | Grueter et al. 2015 |  |
| *Presbytis sabana* | 0 | This study |  |
| *Presbytis siamensis* | 0 | This study |  |
| *Presbytis thomasi* | 0 | Grueter et al. 2015 |  |
| *Procolobus verus* | 0 | Grueter et al. 2015 |  |
| *Propithecus diadema* | 0 | This study |  |
| *Propithecus edwardsi* | 0 | This study |  |
| *Propithecus coronatus* | 0 | This study |  |
| *Propithecus verreauxi* | 0 | Lüpold et al. 2019 |  |
| *Pygathrix nigripes* | 5 | Grueter et al. 2015 | Whiskers/goatee longer in males (1). Scrotum bluish and penis red (4) |
| *Pygathrix nemaeus* | 3 | Grueter et al. 2015 | Penis is pink and pubic area reddish (3) |
| *Rhinopithecus avunculus* | 5 | Grueter et al. 2015 | Male body fur slightly fuller (1). A hint of light blue in male testes (1). Longer corkscrew hair on male tail (1). Male has a more fully developed ruff around face (2) |
| *Rhinopithecus bieti* | 8 | Grueter et al. 2015 | Longer hairs on back and thighs in males (4). Pelage is much more contrastingly colored in males (4) |
| *Rhinopithecus brelichi* | 12 | Grueter et al. 2015 | Adult males have a white patch of fur between their shoulders (4), red hair on back of their head (1) and white nipples (5). Underside of limbs bright gingery-red in males (2) |
| *Rhinopithecus roxellana* | 8 | Grueter et al. 2015 | Adult male has large, striking ‘lip-warts’ on upper lip at mouth corners; these are rudimentary/ lacking in adult females (5). Male has longer hair on shoulders than female (3) |
| *Saguinus fuscicollis* | 0 | Grueter et al. 2015 |  |
| *Saguinus niger* | 0 | This study |  |
| *Saguinus imperator* | 0 | This study |  |
| *Saguinus midas* | 0 | Grueter et al. 2015 |  |
| *Saguinus oedipus* | 0 | Grueter et al. 2015 |  |
| *Saguinus mystax* | 0 | Grueter et al. 2015 |  |
| *Saguinus nigricollis* | 0 | Grueter et al. 2015 |  |
| *Saimiri oerstedii* | 4 | Grueter et al. 2015 | Fatting of shoulders and arms during mating season (4) |
| *Saimiri sciureus* | 4 | Grueter et al. 2015 | Fatting of shoulders, arms and head during mating season (4) |
| *Sapajus apella macrocephalus* | 0 | This study |  |
| *Sapajus nigritus* | 0 | This study |  |
| *Semnopithecus entellus* | 0 | Grueter et al. 2015 |  |
| *Semnopithecus johnii* | 0 | Grueter et al. 2015 |  |
| *Semnopithecus priam* | 0 | This study |  |
| *Semnopithecus schistaceus* | 1 | Grueter et al. 2015 | Male facial hair longer (1) |
| *Semnopithecus vetulus* | 0 | Grueter et al. 2015 |  |
| *Simias concolor* | 0 | This study |  |
| *Symphalangus syndactylus* | 3.5 | Grueter et al. 2015 | Male has a long, prominent black preputial tuft, resembling a short tail (3.5) |
| *Theropithecus gelada* | 13 | Grueter et al. 2015 | Male has long cape of shoulder hair, lacking in female (5). Male has long hair on top and sides of head, lacking in female (5). Male has an hourglass-shaped patch of red chest skin; female’s chest patch is morphologically different, with a fringe of small whitish vesicles (3) |
| *Trachypithecus auratus* | 0 | This study |  |
| *Trachypithecus crepusculus* | 0 | This study |  |
| *Trachypithecus cristatus* | 0 | Grueter et al. 2015 |  |
| *Trachypithecus geei* | 0 | Grueter et al. 2015 |  |
| *Trachypithecus leucocephalus* | 0 | Grueter et al. 2015 |  |
| *Trachypithecus obscurus* | 0 | Grueter et al. 2015 |  |
| *Trachypithecus pileatus* | 0 | Grueter et al. 2015 |  |
| *Trachypithecus pliocephalus* | 0 | This study |  |
| *Varecia variegata* | 0 | Lüpold et al. 2019 |  |

**References**

Dixson, A. F., Dixson, B. J., & Anderson, M. J. (2005). Sexual selection and the evolution of visually conspicuous sexually dimorphic traits in male monkeys, apes and human beings. *Annual Review of Sex Research*, 16, 1-19.

Grueter, C. C., Isler, K., & Dixson, B. J. (2015). Are badges of status adaptive in large complex primate groups? *Evolution and Human Behavior*, 36, 398-406.

Lüpold, S., Simmons, L. W., & Grueter, C. C. (2019). Sexual ornaments but not weapons trade off against testes size in primates. *Proceedings of the Royal Society B*, 286, 20182542.


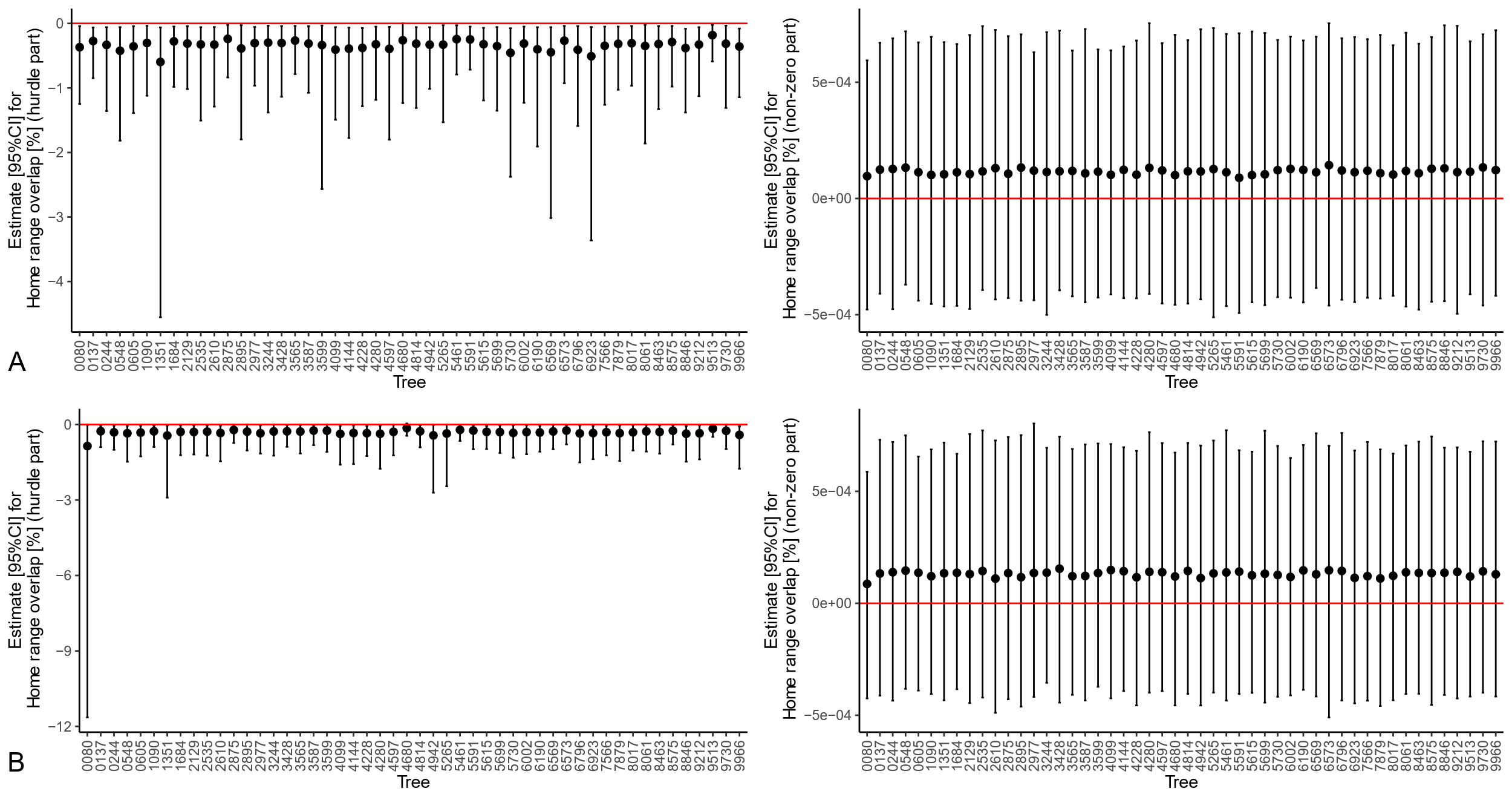


**Figure S1.** Estimates with 95% credible intervals for the effects of percent home range overlap on male ornamentation for each of the 50 MCMC chains using different, randomly selected trees to account for phylogenetic non-independence. The estimates are depicted for the full (A) and conservative (B) ornamentation scores, with left panels representing the hurdle part and the right panels the non-zero part of each model. The red lines highlight the zero threshold.


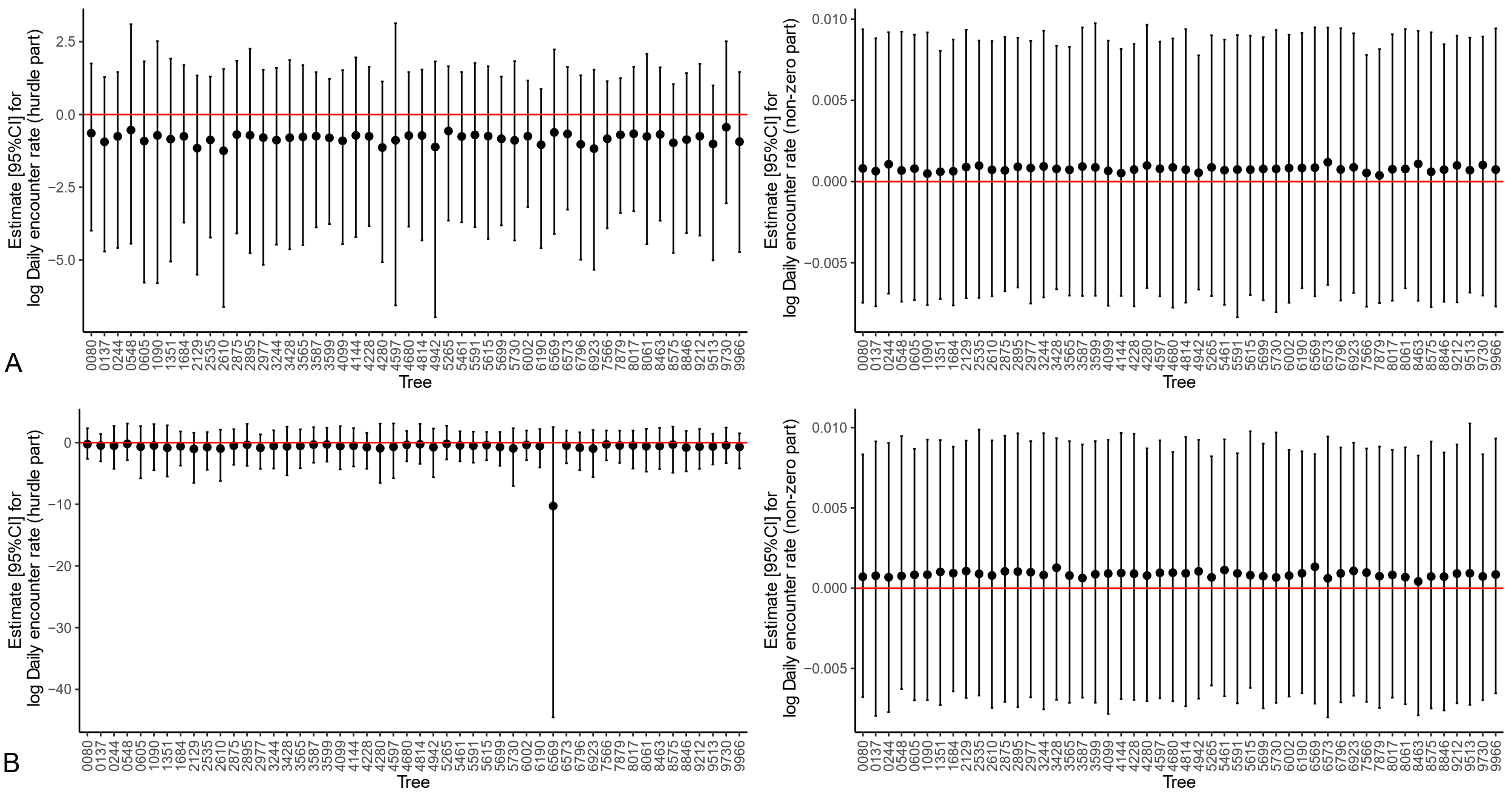


**Figure S2.** Estimates with 95% credible intervals for the effects of the (log-transformed) daily encounter rate on male ornamentation for each of the 50 MCMC chains using different, randomly selected trees to account for phylogenetic non-independence. The estimates are depicted for the full (A) and conservative (B) ornamentation scores, with left panels representing the hurdle part and the right panels the non-zero part of each model. The red lines highlight the zero threshold.


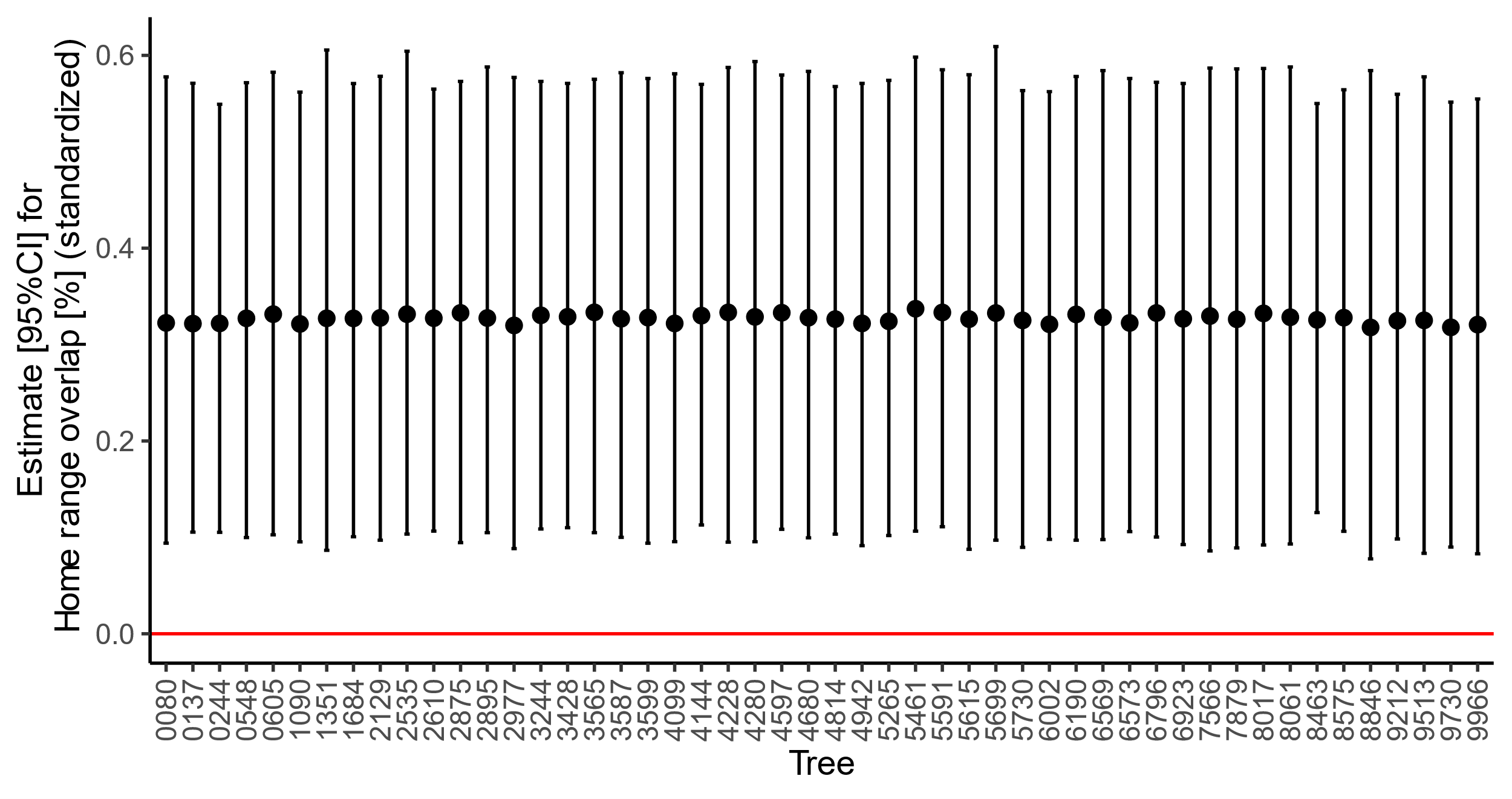


**Figure S3.** Estimates with 95% credible intervals for the effects of percent home range overlap on daily encounter rates between groups for each of the 50 MCMC chains using different, randomly selected trees to account for phylogenetic non-independence. The red lines highlight the zero threshold.


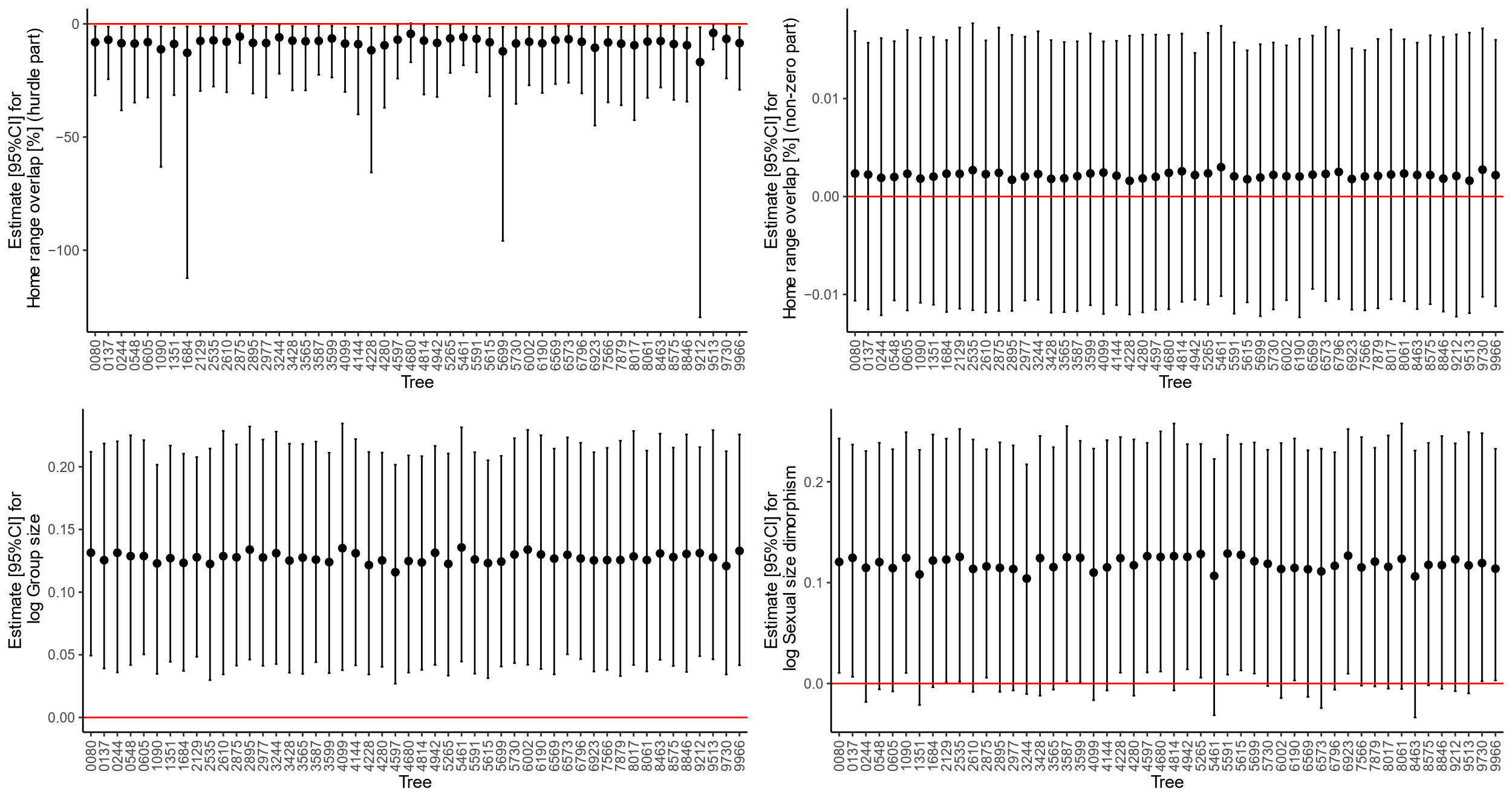


**Figure S4.** Estimates with 95% credible intervals for the effects of percent home range overlap (top panels, hurdle and non-zero parts of the model), group size and sexual size dimorphism (both log-transformed, bottom panels) on male ornamentation for each of the 50 MCMC chains using different, randomly selected trees to account for phylogenetic non-independence. The red lines highlight the zero threshold.


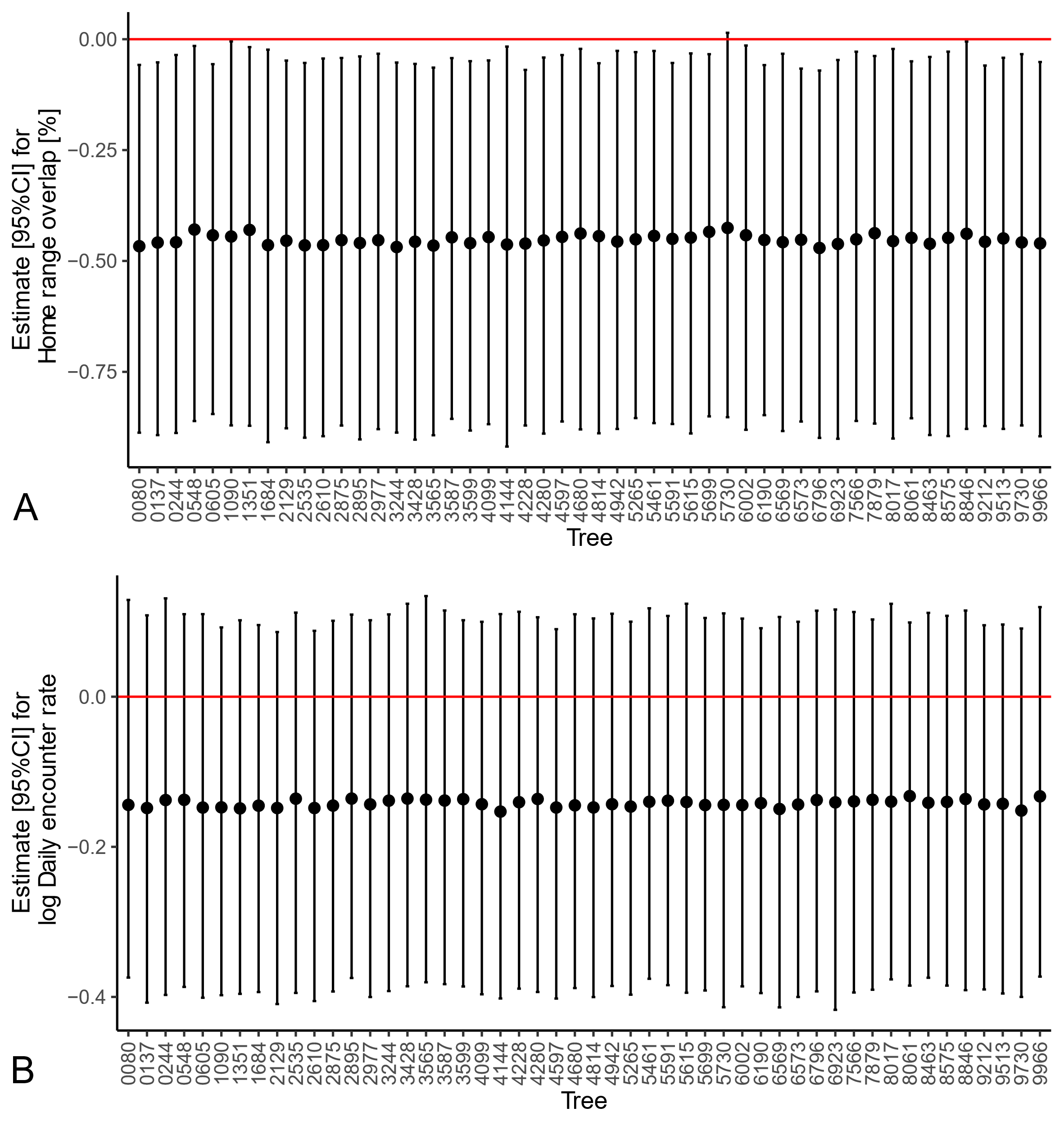


**Figure S5.** Estimates with 95% credible intervals for the effects of percent home range overlap (A) and daily encounter rate between groups (B) on the proportion of agonistic encounters for each of the 50 MCMC chains using different, randomly selected trees to account for phylogenetic non-independence. The red lines highlight the zero threshold.
